# A public health perspective of SARS-CoV-2 evolution and surveillance strategies in Germany from 2020 to 2023

**DOI:** 10.1038/s43856-025-01093-1

**Published:** 2025-11-11

**Authors:** Djin-Ye Oh, Martin Hölzer, Daniela Börnigen, Sofia Paraskevopoulou, Susanne Duwe, Matthias Budt, Romy Kerber, Agata Mikolajewska, Sindy Böttcher, Janna Seifried, Walter Haas, Ralf Dürrwald, Stephan Fuchs, Stefan Kröger, Max von Kleist, Thorsten Wolff, Martin Mielke

**Affiliations:** 1 Influenza and Other Respiratory Viruses (FG17), Robert Koch Institute, Berlin, Germany; 2 Genome Competence Center (MF1), Robert Koch Institute, Berlin, Germany; 3 Systems medicine of infectious diseases (P5), Robert Koch Institute, Berlin, Germany; 4 Respiratory Infections (FG36), Robert Koch Institute, Berlin, Germany; 5 Strategy and Incident Response (ZBS7), Robert Koch Institute, Berlin, Germany; 6 Gastroenteritis and Hepatitis Pathogens and Enteroviruses (FG15), Robert Koch Institute, Berlin, Germany; 7 Infectious Disease Epidemiology (Dept. 3), Robert Koch Institute, Berlin, Germany; 8 Department of Mathematics and Computer Science, Freie Universität Berlin, Berlin, Germany; 9 Infectious Diseases (Dept. 1), Robert Koch Institute, Berlin, Germany; 10 Helmholtz Institute for One Health, Helmholtz-Centre for Infection Research, Greifswald, Germany; 11 Hospital Hygiene, Infection Prevention and Control (FG14), RKI, Berlin, Germany; 12 Highly Pathogenic Viruses (ZBS1), RKI, Berlin, Germany; 13 Methods Development and Research Infrastructure (Dept. MFI), RKI, Berlin, Germany

**Keywords:** Viral infection, SARS-CoV-2, Viral epidemiology

## Abstract

This review summarizes key virological parameters of SARS-CoV-2, the clinical spectrum of COVID-19, antiviral options, resistance, and the evolution of SARS-CoV-2 during the first four years of the pandemic. It draws on evidence that has been continuously updated throughout the pandemic by the interdisciplinary working group ‘SARS-CoV-2 Diagnostics and Evolution’ at Robert Koch Institute (RKI), Germany’s national public health institute. We describe basic SARS-CoV-2 characteristics and highlight notable virus variants from 2020 to mid-2023. During this period, the nationwide collection of SARS-CoV-2 genomes provided a substantial resource for monitoring viral lineage frequencies and mutations. We summarize this dataset to underscore the importance of virological surveillance in the context of public health and pandemic preparedness.

## Abstract (web summary)

Oh, Hölzer et al., discuss the multidisciplinary approach used for monitoring the genomic and virological evolution of SARS-CoV-2 during the COVID-19 pandemic and its implications on public health in Germany. They emphasize the need for continuous surveillance and adaptation of public health strategies to address evolving viral threats.

## Introduction

SARS-CoV-2 (severe acute respiratory syndrome coronavirus type 2) is a coronavirus (genus: *Betacoronavirus*, subgenus: *Sarbecovirus*) identified in early 2020 as the causative agent of COVID-19^1^. Coronaviruses are widely distributed among mammals and birds. They are classified in the *Coronaviridae* family of RNA viruses (realm: *Riboviria*, order: *Nidovirales*, suborder: *Cornidovirineae*), in which the large subfamily *Orthocoronavirinae* includes four genera: *Alpha-*, *Beta-*, *Gamma-*, and *Deltacoronavirus*. Because of their capacity for homologous recombination, coronaviruses can relatively easily expand their host range and overcome cross-species boundaries^2^. The seven known human pathogenic coronaviruses (HCoV) fall into two genera: *Alphacoronavirus* (HCoV-229E, HCoV-NL63) and *Betacoronavirus* (HCoV-HKU1, HCoV-OC43, MERS-CoV, SARS-CoV, SARS-CoV-2). The viruses HCoV-229E, HCoV-NL63, HCoV-HKU1, and HCoV-OC43 are known as seasonal or endemic coronaviruses and primarily cause mild colds. However, in early childhood, the elderly, and immunocompromised individuals, severe cases of pneumonia can occur^3^. SARS-CoV, MERS-CoV, and SARS-CoV-2 have only recently spilt over from animal reservoirs to humans^4^. Infections with these “*emerging pathogens*” can cause severe disease with fatal outcomes.

**Fig. 1:**
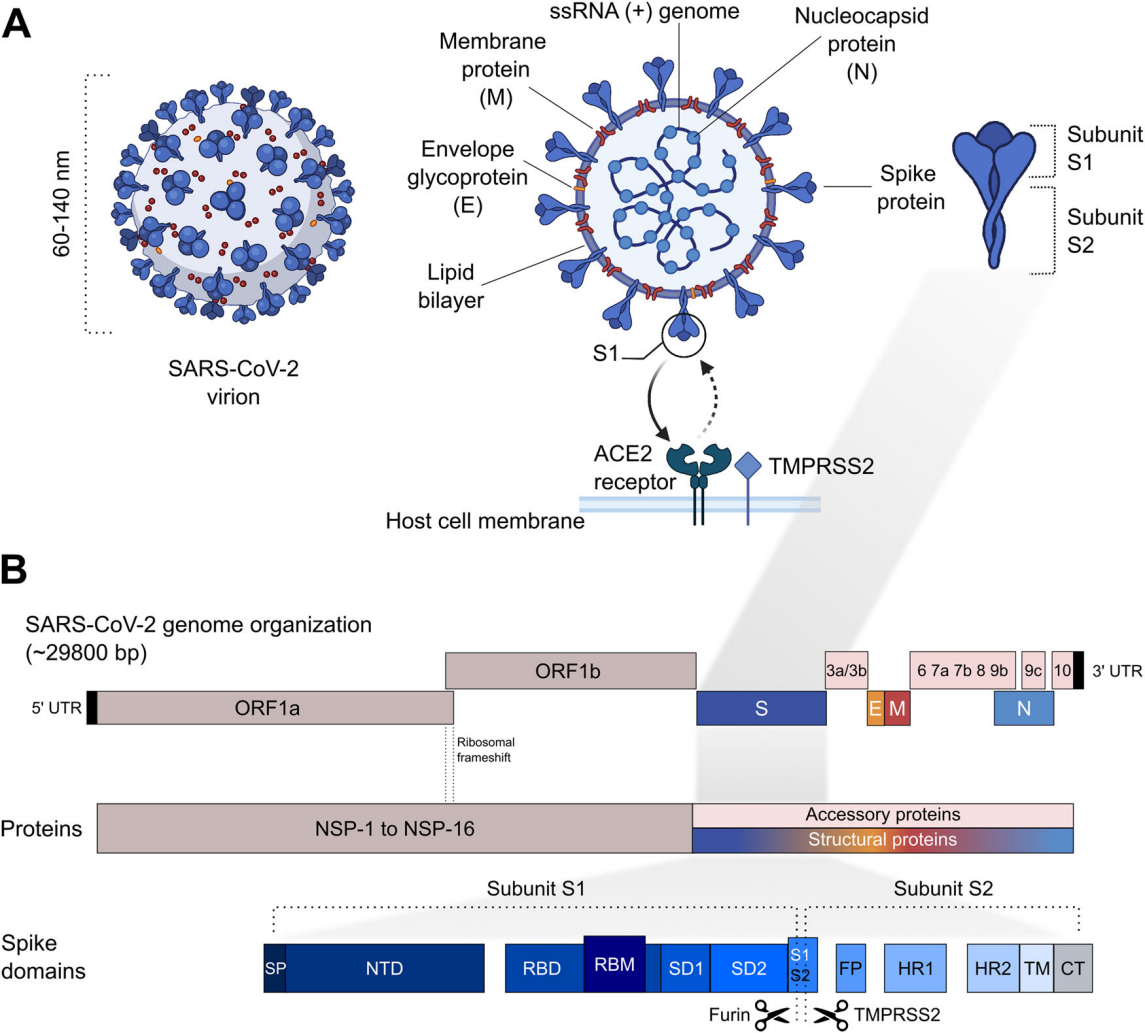
Structure of SARS-CoV-2 virion and genome organization. **A** Schematic of the SARS-CoV-2 virion showing the lipid bilayer envelope containing membrane (M) and envelope (E) proteins, spike (S) proteins with subunits S1 and S2, and the nucleocapsid (N) protein bound to the positive-sense single-stranded RNA (ssRNA (+)) genome. The spike protein engages the host ACE2 receptor and is primed by TMPRSS2. **B** Genome organization of SARS-CoV-2 (~29,800 bp), including open reading frames ORF1a and ORF1b encoding nonstructural proteins (NSP1–16), structural proteins (S, E, M, N), and accessory proteins. The spike (S) protein is further divided into domains, including signal peptide (SP), N-terminal domain (NTD), receptor-binding domain (RBD) with receptor-binding motif (RBM), subdomains (SD1, SD2), fusion peptide (FP), heptad repeats (HR1, HR2), transmembrane domain (TM), and cytoplasmic tail (CT). Cleavage sites for furin and TMPRSS2 are indicated. The figure combines published information and was adapted accordingly from refs. ^253–256^. Figure elements in **A** were created with BioRender.com.

Betacoronaviruses are membrane-enveloped RNA viruses and form virions approximately 60-140 nm in diameter with large (20-25 nm long) surface glycoproteins (spikes)^5^ (Fig. 1). They have a single-stranded RNA genome of positive polarity that is about 30 kilobases long, making it one of the largest genomes of all known RNA viruses. The genome encodes 29 proteins, including 16 non-structural, four structural, and nine accessory proteins which are involved in various steps of the virus’ life cycle^6^. The non-structural proteins are responsible for RNA replication. The structural proteins are the spike glycoprotein (S), the envelope small membrane protein (E), the membrane protein (M), and the nucleoprotein (N). The S, E, and M proteins are incorporated into the viral membrane that envelops the nucleocapsid, which is composed of the N protein and the viral genome^3^. The S (or spike) protein is responsible for entering the host cell and consists of two subunits. The S1 subunit contains the Receptor Binding Domain (RBD), which binds to the host cell receptor, and an amino-terminal (N-terminal) domain (NTD). The RBD contains the receptor-binding motif (RBM), which, together with the NTD, is the main target of neutralizing antibodies^7^. After host cell receptor binding, the S2 subunit mediates the fusion of the viral envelope and host cell membrane and the subsequent viral RNA release into the cytoplasm^7^. Neutralizing antibodies directed against epitopes on the RBD and the NTD inhibit cell entry of the virus and are one of the strongest available correlates of vaccine-induced protection against SARS-CoV-2 infection^8–10^. Thus, many vaccines utilize exclusively the spike protein as antigenic component^11–13^. SARS-CoV-2, like SARS-CoV and HCoV-NL63, engages the transmembrane angiotensin-converting enzyme 2 (ACE2) of host cells as a receptor, enabling subsequent entry into the host cell^7^: The spike protein of the virus attaches to the ACE2 receptor on the host cell and undergoes proteolytic activation. This activation involves cleavage at the S2’ site, facilitated by TMPRSS2 on the cell surface or by endosomal cathepsins within the cell. This process transitions the spike protein into a metastable state, allowing the viral and host cell membranes to fuse and initiate infection^14,15^. ACE2 and TMPRSS2 are co-expressed at high levels in the nasal epithelium, which may explain the efficient spread and shedding of SARS-CoV-2 in the upper respiratory tract^16^. High ACE2 density has been reported not only in the respiratory tract but also, for example, on enterocytes, vascular endothelial cells, renal epithelium, and myocardial cells^17–21^. Histopathological studies have demonstrated SARS-CoV-2 organ tropism for the lung, intestine, kidney, heart, and the central nervous system (CNS) (see^22–26^).

In contrast to pre-Omicron variants, SARS-CoV-2 variants of the Omicron complex tend to use the endosomal entry pathway mediated by cathepsins L/B, rather than the membrane (ACE2) entry pathway mediated by TMPRSS2^27,28^. Cathepsins are located in the endosome and the cleavage of cathepsin occurs endosomally when the virus-ACE2 complex is internalized via clathrin-mediated endocytosis into the late endolysosomes^15^.

The emergence of the COVID-19 pandemic coincided with the advent of extensive global sequencing and data-sharing capabilities. This created an unprecedented opportunity to monitor the adaptive evolution of a respiratory RNA virus in near real-time on a global scale. It allowed us to monitor the virus while it developed the capacity to circulate in the human population and transmit among individuals with prior immunological exposure^29,30^.

A comprehensive understanding of the challenges posed by SARS-CoV-2, the virus responsible for the COVID-19 pandemic, requires close collaboration among virologists, bioinformaticians, clinicians, epidemiologists, and public health experts. At Robert Koch Institute, the national public health institute of Germany, an interdisciplinary team has continuously reviewed evidence on several critical aspects of COVID-19, including diagnostic and clinical features as well as viral evolution throughout the first years of the pandemic. This review outlines data obtained from 2020 to 2023 as a result of these concerted efforts, emphasizing evolutionary aspects, especially the rise of virus variants such as the Omicron complex and their impact from a public health perspective. It provides data to illustrate the ongoing need for robust surveillance systems to detect changes in respiratory virus circulation, guiding clinical and public health decisions.

## SARS-CoV-2 variant nomenclature

Based on their mutation profiles, SARS-CoV-2 genetic variants are classified into clades as provided by efforts such as Nextclade^31^, or into lineages by systems such as Pangolin^32^, which also considers epidemiological criteria including localized and temporally clustered occurrences. The terms “lineage” and “variant” are used interchangeably and both refer to viruses which differ in genome sequence by several mutations. A “sublineage” is a lineage descendant, i.e., it harbors the same mutations as the “parental lineage” in addition to its distinct mutations. Eventually, all lineages (or variants) with their sublineages are placed in a large genealogy of SARS-CoV-2, which is maintained by the Pangolin community^32^.

The effects of specific mutations on the phenotypic properties of the virus, such as transmissibility, virulence, or immunogenicity, are subject to intense investigations, as a firm understanding will allow more reliable estimates of the risks posed by emerging variants [summarized in refs. ^33,34^]. If these properties of the virus change, then the term “strain” is used to differentiate this new variant from the others.

WHO classifies variants of SARS-CoV-2 based on their potential impact on global health and, thus, the degree of surveillance efforts required for each variant^35,36^. A Variant Under Monitoring (VUM) is a SARS-CoV-2 variant with genetic changes potentially affecting viral characteristics that shows early signs of growth (transmission) advantage, necessitating increased monitoring. A Variant of Interest (VOI) features genetic alterations that affect viral properties, demonstrating a growth advantage in multiple WHO regions or significant epidemiologic impact, signaling a threat to global health. The highest risk is assigned to a Variant of Concern (VOC), which not only meets VOI criteria but additionally leads to higher disease severity, affects COVID-19 epidemiology significantly, or markedly reduces vaccine protection against severe disease requiring major public health interventions.

Throughout the pandemic, the WHO has categorized five SARS-CoV-2 variants as VOCs: B.1.1.7, B.1.351, P.1, B.1.617.2, and B.1.1.529^37,38^. In line with the simplified WHO supplementary nomenclature, these are also called Alpha, Beta, Gamma, Delta, and Omicron according to Greek letters in the order of discovery^35^. The characteristics of the pre-Omicron VOCs and the Omicron complex are described in more detail in Boxes 1–4.

Until March 2023, all Omicron sublineages within the Omicron complex were classified as VOCs. However, this automatic inheritance of the parental VOC designation limited the ability to categorize specific Omicron sublineages and sub-sublineages as VOC/VOI/VUMs themselves. Therefore, this classification system did not provide the resolution needed to differentiate new and phenotypically distinct sublineages from other variants, including the parental Omicron lineages (e.g. BA.1, BA.2). To better track the evolution of SARS-CoV-2 variants, the WHO announced on March 15, 2023, that notable Omicron sublineages could be classified as VUM or VOI, rather than being grouped under the Omicron VOC label^36^. The original Omicron parent lineage (B.1.1.529) was de-escalated and is now classified as a “formerly circulating VOC” along with Alpha, Beta, Gamma, and Delta. Consequently, current Omicron variants lack the VOC label, even though they are not considered less dangerous than the B.1.1.529 parent lineage. The purpose of “resetting” the naming system was to enable appropriate naming and tracking of future sublineages that may be even more dangerous than the currently circulating ones. The declaration of a variant as VOC is still reserved for significant SARS-CoV-2 variants that are given a Greek letter name (e.g., Alpha, Delta, Omicron).

BOX 1: Index virus and D614G **Index virus**. The initial virus variant that was detected in the first SARS-CoV-2 outbreak in Wuhan (GenBank accession number: NC_045512.2)^257^. Variants of concern evolved from this original virus, which entered the human population in 2019. This virus is also termed “wild type”. As it is the original virus and predecessor, and because the different WHO alert levels for variants did not exist in 2019, it has not been designated as a variant of concern.

**D614G**. The epidemic success of 614G variants is attributed to increased intrinsic transmissibility. Mechanistic studies underpinning this explanation demonstrated a change in the spike protein region towards a more open conformation, which favors binding to the ACE2 receptor protein of target cells^258^. This resulted in higher in vitro infectivity (lower infectious dose) of 614G viral particles^259^, which replicate more effectively than D614 viral particles, especially in nasal epithelial cells^260,261^. This is also associated with higher transmission rates in animal experiments^260^ and higher viral loads in clinical samples from the upper respiratory tract. However, with higher transmissibility, there is no evidence of more severe clinical courses in humans^58,262^.

BOX 2: Pre-Omicron VOCs: Alpha and Delta Alpha (B.1.1.7; 501Y.V1). Beginning in mid-December 2020, increasing spread of this variant, initially designated as SARS-CoV-2 VOC 202012/01, was reported from the United Kingdom^263,264^. The variant, assigned to lineage B.1.1.7 (Alpha, 501Y.V1) displayed numerous non-synonymous polymorphisms. For several of these amino acid substitutions, there were theoretical and experimental indications of phenotype effects, including the ORF Q27stop mutation resulting in the effective loss of ORF8, and the Spike mutations P681H, adjacent to the furin cleavage site, and N501Y, known to increase receptor affinity and viral fitness^88,90,91^.

Infections with Alpha were associated with more severe illness, indicating higher pathogenicity^96–98,265^. Epidemiological and phylodynamic data/modeling implied an approximately 1.5-fold higher reproduction number^95,124,266^, in line with contact tracing data indicating a higher rate of infected contacts^267^. Therefore, Alpha was thought to have higher intrinsic transmissibility than previous lineages. Among the causative mechanisms discussed were higher viral loads found in several^268,269^ but not all^270,271^ studies, higher ACE2 receptor affinity of the Alpha spike protein, which had been observed for the isolated N501Y polymorphism in vitro^90,91^ and enhanced resistance of the Alpha spike to premature proteolytic cleavage in the extracellular environment of the respiratory tract^272^.

Alpha became the predominant variant in the first half of 2021, not only in the UK but also in many other countries^37,59,273,274^.

Delta (B.1.617.2). In May 2021, WHO declared the SARS-CoV-2 lineage B.1.617.2 (Delta), which had first been detected in India in October 2020, a VOC, based on clear epidemiologic evidence of increased transmissibility gathered in the UK: First, Delta cases rose at higher rates than the previously dominant Alpha variant. Second, contact tracing data showed that Delta-infected individuals had a higher proportion of infected contacts^275^. Based on higher transmissibility of Delta over Alpha and of Alpha over the index virus, the baseline reproduction number (R0) of Delta was estimated to range between 6 and 7105. Higher hospitalization rates, ICU admissions, and deaths were observed for Delta compared to Alpha infections, implying higher severity^276–278^.

The Delta spike carries several polymorphisms, including L452R and P681R234. The isolated L452R substitution enhances ACE2 receptor affinity and infectivity^279^ and appears to affect antigenicity^275,279^. The P681R substitution is implicated with increased proteolytic cleavage of the spike protein at the S1/S2 site, potentially promoting replication^280^. Indeed, consistent with higher intrinsic transmissibility, Delta showed greater replication efficiency than Alpha in relevant in-vitro models, with the Delta spike predominantly in its cleaved form, unlike Alpha. The Delta spike also facilitated more syncytium formation. These laboratory findings aligned with multiple observations of increased clinical severity suggesting higher pathogenicity for Delta^281–285^.

Delta demonstrated reduced susceptibility to neutralization by both convalescent and vaccine sera, indicating immune evasion^281,286,287^. In line with the laboratory data, observational clinical data showed reduced vaccine protection against symptomatic Delta infections in individuals who had received only one vaccine dose^288–290^. Thus, the fitness advantage underpinning Delta’s evolutionary success was based on higher intrinsic fitness and its capacity to partially evade prior immunity.

BOX 3: Pre-Omicron VOCs with pronounced immune escape: Beta and Gamma Beta (B.1.351; 501Y.V2). In December 2020, genomic surveillance in South Africa revealed the increasing prevalence of a SARS-CoV-2 variant (B.1.351, Beta, 501Y.V2) with numerous nonsynonymous spike mutations [L18F, D80A, D215G, R246I, K417N, E484K, N501Y, D614G, A701V], including three amino acid substitutions in the RBD region (K417N, E484K, and N501Y)^92^. Both the K417N and the E484K polymorphism were known to decrease the sensitivity of the virus to neutralizing antibodies. This suggested that the Beta growth advantage might be due to immune evasion, namely that the immune response against Beta (B.1.351) induced by infection or vaccination with the index virus is less effective^291–294^. Indeed, the neutralizing activity of convalescent and vaccine plasma against this variant was reduced^291,295,296^. Moreover, reinfections with Beta were common, as indicated by the fact that COVID-19 incidence in seropositive individuals was similar to that observed in seronegative individuals^297^, and phase 3 clinical studies indicated reduced vaccine efficacy against this variant^298^. Although certain vaccines, namely RNA vaccines retained reasonable levels of effectiveness^167^, Beta-adjusted vaccines were developed and assessed in vivo^299^. While the main fitness advantage of Beta is its ability to evade humoral immunity, facilitating reinfection and vaccine breakthroughs to increase effective transmissibility, higher intrinsic transmissibility has also been discussed^92,300^, based on in vitro data indicating enhanced ACE2 receptor affinity when the E484K and N501Y polymorphisms occur in combination^91^. Before Omicron, Beta was the SARS-CoV-2 variant that was the most antigenically distinct from the index strain. Gamma (P.1; 501Y.V3). A SARS-CoV-2 variant descending from lineage B.1.1.28, designated P.1 (Gamma, 501Y.V.3), was noted in travelers returning from Brazil to Japan in January, 2021. Gamma’s earliest detection was in samples collected from COVID-19 patients in Manaus, the capital of Amazonas state in December 2020, where this new VOC increased rapidly and drove a massive surge of infections, hospitalizations, and excess deaths^93,94,301,302^. Notably, the Gamma wave of Manaus occurred in a setting of 76% seroprevalence, indicating that most citizens had been infected previously, namely during the first COVID-19 wave that had spread practically unmitigated through the capital^303^. Gamma displayed multiple spike polymorphisms [L18F, T20N, P26S, D138Y, R190S, K417T, E484K, N501Y, D614G, H655Y, T1027I, V1176F] and resembled the Beta (B.1.351) variant in key RBD positions (K417, E484, N501). Like Beta, Gamma showed partial resistance against convalescent and vaccine sera, indicating that immune evasion, facilitating transmission to and replication in previously infected hosts, represented its key fitness advantage. These observations challenged the then-widely-held theory that natural herd immunity would bring the pandemic to an end^94,301,304^.In addition to immune evasion, higher intrinsic transmissibility, and increased pathogenicity were also discussed for this variant, which brought about a healthcare system collapse in Manaus^93,301,302^.

BOX 4: The Omicron complex of variants On November 26th, 2021, WHO declared the new SARS-CoV-2 lineage B.1.1.529 to be a VOC, designated “Omicron”106. Phylogenetic studies demonstrated pronounced divergence from other variants with approximately 50 amino acid substitutions relative to the index virus, more than half of which were localized in the spike protein. Several of these amino acid changes (spike: K417N, S477N, E484A, N501Y, D614G, H655Y, P681H) were known to affect phenotype^90,91,292–294^. Since then, Omicron has diversified into multiple sublineages whose geographic distribution varies^305–307^. The first Omicron lineages, BA.1 and BA.2, were soon replaced by BA.5 and its close relative BA.4 (sometimes collectively referred to as BA.4/5 due to identical spike amino acid sequences). Subsequently, a variety of Omicron sublineages emerged, many of which displayed identical mutations that had evolved convergently, including BQ.1* (a BA.5 sublineage with additional amino acid substitutions impacting critical antigenic epitopes, e.g., N460K, K444T, and R346T)^308^ and XBB* (which had emerged from recombination of the sublineages BJ.1 and BA.2.75*). In vitro data indicated distinct immune escape properties^69,309^. The designation of Omicron sublineages follows the Pangolin nomenclature system^32,310^: A lineage designation may contain a maximum of three numbers separated by points (e.g., “BA.5.3.1”). If a designated lineage with three numbers diversifies further, the next available letter (or letter combination) is assigned to the corresponding sublineage (https://github.com/cov-lineages/pango-designation/issues/). For example, Omicron BA.579^125^, diversified into multiple sublineages and new letters were needed to designate a subsequent sublineage, e.g. BE.* or BF.* (BE.1 represented an alias for BA.5.3.1.1, and BF.1 represented an alias for BA.5.2.1.1).

Immune evasion. Antigenic cartography analyses demonstrate that Omicron is antigenically distinct from the index virus and all previous VOCs111^311–313^,. This finding has been corroborated with broad clinical and epidemiological evidence of reinfections and vaccine breakthroughs, which also shows that (index) vaccine protection against symptomatic infection is reduced but not completely abolished; Omicron is therefore not a complete immune escape mutant^109,314–321^. Emerging Omicron lineages displayed marked evasion of the humoral immunity induced by older Omicron lineages, indicating antigenic drift-like evolution within the Omicron complex^70^. Mono- and bivalent vaccines based on the index strain appeared to boost antibodies against the few conserved Spike epitopes present in the index strain as well, indicating immune imprinting, where prior exposure to ancestral virus might negatively influence the immune response to subsequent virus variants^70,322–324^. Therefore, since early 2023, COVID-19 vaccines are monovalent and based on Omicron XBB.1.5^325,326^.

Clinical manifestation and disease severity. Epidemiological and clinical evidence indicates that compared to previous variants, Omicron infections are associated with less severe illness, affecting the upper more than the lower respiratory tract and leading to fewer hospitalizations^146,147,305,314,327–332^. Some authors found similar clinical severity of Omicron in unimmunized elderly and children, indicating that the observed lower severity of Omicron is not only due to lower intrinsic pathogenicity but also to higher population immunity levels^188–190,305^.

## Mechanisms of SARS-CoV-2 evolution

Several factors, including the intrinsic mutation rate, virus and host biology, infection rates and selection pressures that have changed (and continue to vary) over the course of the pandemic, contribute to the evolution of the virus. Changes in the viral genome occur during the replication of RNA viruses in an infected host and can be transferred to the viral progeny, which are then subject to the immune selection pressure of their respective hosts. Although the RNA polymerase of coronaviruses has a rudimentary proofreading function that reduces such replication errors, SARS-CoV-2 can still accumulate a significant number of nucleotide polymorphisms. Additionally, recombination may occur when two genetically distinct viruses infect the same cell. However, *within-host* evolution of the virus does not directly translate into an observable *between*-*host* evolution^39^. Due to the short infectious period of SARS-CoV-2 (a few days)^40^, immunocompetent individuals typically transmit virus particles with minimal genetic variation compared to the initial infection. Outbreak genetic analyses have revealed that virus genomes obtained from transmission pairs (i.e., SARS-CoV-2 cases with an epidemiological link suggesting direct transmission) are identical for most pairs^41–43^. This attribute of SARS-CoV-2 biology contributes to the fact that the evolutionary rate of the virus is orders of magnitude lower than would be expected given its RNA polymerase error rate. However, high infection rates, or a prolonged course of infection may accelerate the overall rate of evolution despite the low rate of within-host evolution^44^. Importantly, infections that persist for months, occasionally observed in the context of immunocompromised individuals (so-called: long-shedders), may result in a vast array of mutant viruses with new capabilities^45–47^. While some of these highly evolved variants may be transmitted^47^, ongoing debate surrounds the extent to which long-term infections, particularly in immunocompromised patients, contribute to directed virus evolution^46,48–52^. This aspect remains a critical area of study in understanding the ability of the virus to adapt^53^.

### Evolutionary rates and mutations

**Fig. 2:**
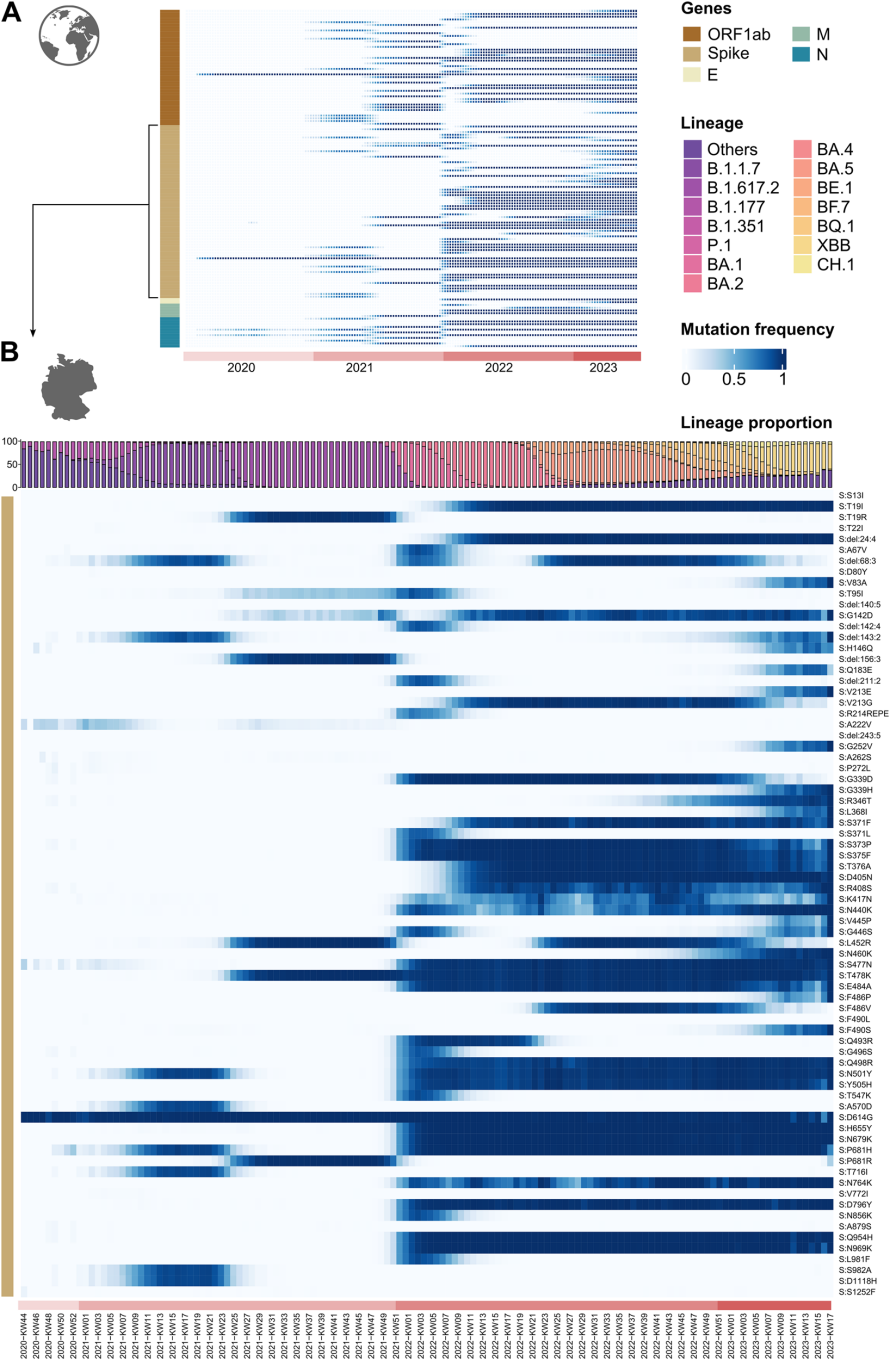
Mutation frequencies in the SARS-CoV-2 genome throughout the pandemic and independent of lineage assignments, based on published sequence data. Amino acid (aa) substitutions shown here have exceeded a frequency of 70% in at least one calendar week (see Supplementary Text T1 for details). **A** The global view is based on more than 8.5 million genomes from GISAID and illustrates the relative occurrence of mutations within selected viral genes over time. Note that genes, signified by the vertical bar to the left are not depicted to scale; i.e., the density of amino acid substitutions accumulated over the spike gene ( ~ 3600 nt) is considerably higher than that accumulated over ORF 1a/b (>15,000 nt). **B** Mutation frequencies within the spike gene, resulting in amino acid replacements during any given calendar week, are depicted for the German genomic surveillance data set; to avoid sampling bias, only those 360,800 genomes were included that had been marked as “randomly sampled” by the submitter (see Supplementary Methods for detailed explanation). The abundance of selected major lineages circulating in Germany between the end of 2020 and April 2023 is visualized at the top of the heatmap. Data used for this visualization is part of the German SARS-CoV-2 genomic surveillance data set collected from the German Electronic Sequence Data Hub (DESH)^59^, published at ref. ^60^.

The evolutionary rate, or substitution rate, measures how fast detectable mutations accumulate in a viral population. Unlike the mutation rate, which includes all new mutations, the evolutionary rate focuses on those reaching significant frequencies^44^. Evolutionary rates vary among genes, in part due to heterogeneous selection pressures. Indeed, for SARS-CoV-2 evolutionary rates vary not only by genomic region but also by phase of the pandemic^44^. Here, Fig. 2A provides insight into the evolutionary rates over different SARS-CoV-2 genomic regions, based on over 8.5 million viral genomes obtained between January, 2020 and June, 2023^54–57^ (for more details see Supplementary Text T1): During the first year of the pandemic, only few amino acid substitutions became fixed, indicating an overall low evolutionary rate. The two amino acid substitutions that did gain rapid predominance in early 2020, S D614G and, as part of the same haplotype, RdRp P323L (encoded by ORF1a/b) were associated with more transmissions, potentially reflecting adaptation to the human host^58^. In subsequent years, with rising case and transmission numbers, many more amino acid substitutions have accumulated.

The viral gene accumulating the highest number of nonsynonymous mutations relative to its length was the spike gene. This implies that the evolutionary rate increased over the course of the pandemic and was highest over the spike gene. In other words, this SARS-CoV-2 gene evolved the fastest, particularly once widespread infections and vaccination campaigns led to the development of population immunity. A more detailed view into the rapid accumulation of spike amino acid substitutions is provided in Fig. 2B, which visualizes a portion of the German SARS-CoV-2 genomic surveillance dataset^59,60^. The observation that the spike gene has the highest evolutionary rate comes as no surprise: the spike protein is the target of all neutralizing antibodies induced by vaccines and infection. Mutations in the spike gene can confer resistance to neutralization by vaccine- or infection-induced immune responses (“immune evasion”, “immune escape”), facilitating breakthrough infections and transmissions from immunized individuals^61^. Higher immune selection pressure (due to the global increase in population immunity) promotes antigenic drift in the spike gene, enhancing immune escape. The high structural plasticity of the Spike RBD is particularly conducive to the emergence of *escape variants*^62^. Moreover, Spike amino acid changes can influence viral infectivity and replication capacity, e.g. by increasing ACE2 receptor affinity and the ability for ACE2-independent cell entry^63,64^. This contrasts with the loss of structural stability of the spike trimer associated with some amino acid exchanges^65,66^. The balance of these factors determines the evolution of the Spike protein, a dynamic and complex process where transmissibility and immune escape are the ultimate determinants of positive selection.

**Fig. 3:**
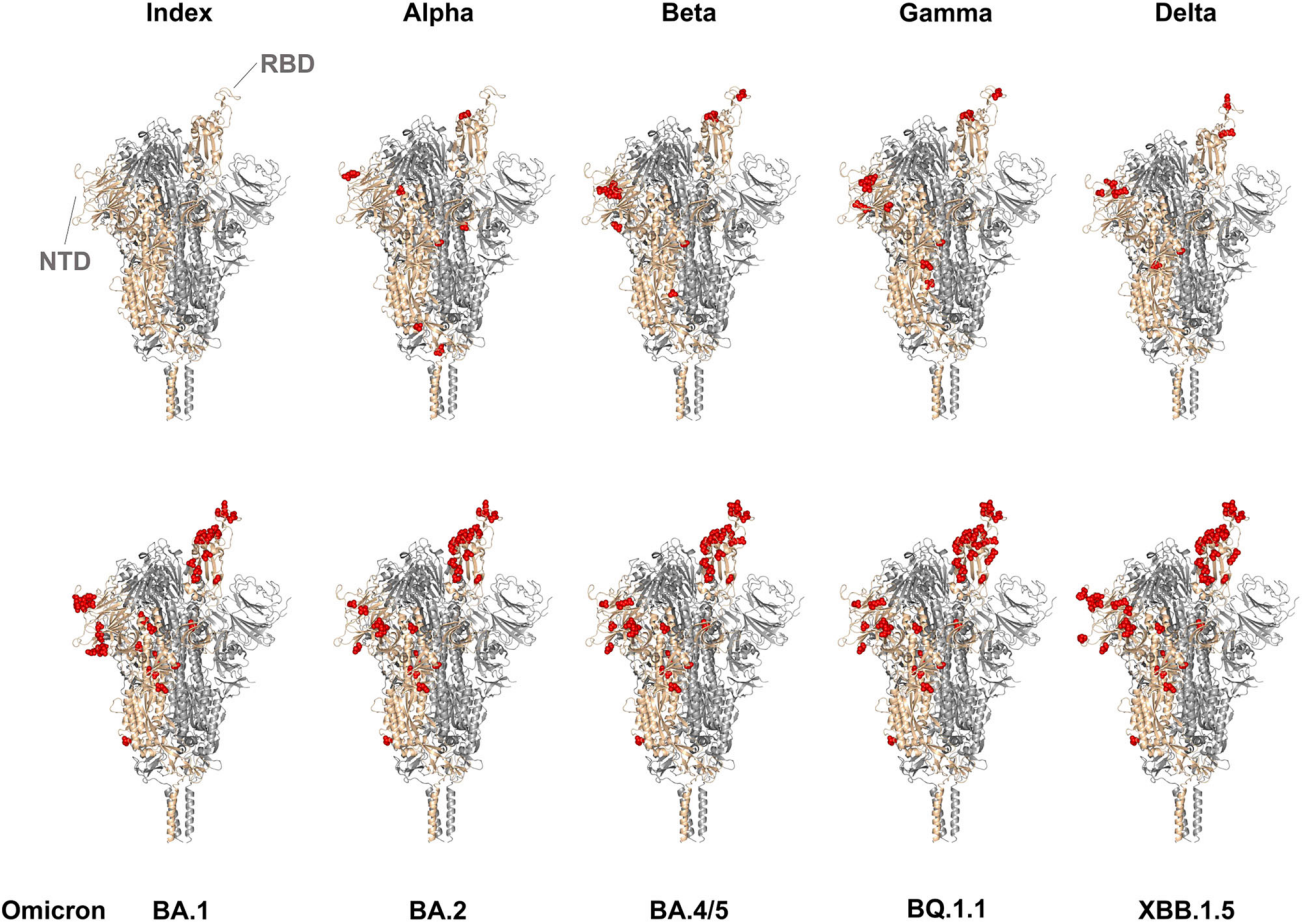
Variant-specific amino acid changes on the 3D structure of the spike (S) protein for the Index (Wuhan), Alpha, Beta, Gamma, Delta, and selected Omicron variants. The receptor-binding domain (RBD) and N-terminal domain (NTD) locations are indicated. Red spots indicate non-synonymous mutations calculated with a mutation frequency of over 70% in genomes of the corresponding lineage based on 8.6 million GISAID samples (see Supplementary Text T1 for details). Here, the 3D structure of the S protein (PDB: 7SBO) was plotted using the PyMOL Molecular Graphics System, Version 2.0 Schrödinger, LLC.

The Omicron variant, which reached global predominance shortly after its emergence in late November 2021^67^, has continuously evolved since and is divided into many sublineages^68^. Despite their genetic diversity, these sublineages display convergent evolution in the Spike protein (mutations independently acquired at the same positions), promoting the emergence of variants that are capable of evading population immunity^69–71^. Figure 3 depicts the spike proteins of selected Omicron sublineages, the index virus, and the pre-omicron VOCs, highlighting the variant-specific spike amino acid changes.

By the end of 2022, the spreading dynamics of sublineages with new escape properties varied from region to region, reflecting the increasing complexity of the global immunity landscape. For example, the closely related Omicron-BA.4/BA.5 variants initially displayed slower spread in many European countries than in South Africa. A plausible explanation is that, unlike in South Africa, many people in Europe were infected with Omicron-BA.2 before the arrival of BA.4/5. Omicron-BA.2 displays high antigenic similarity with Omicron-BA.4/5. Therefore, after a pronounced Omicron-BA.2 wave, it can be assumed that population immunity against Omicron-BA.4/BA.5 reaches high levels. Thus, the course of waves with new (sub-)variants depends on which variants have dominated the preceding waves at a given time in a given region^61^.

While the Spike protein is arguably most relevant to the adaptive evolution of the virus in humans, mutations in other genomic regions are also selected (Fig. 2A and Supplementary Fig. S1). They may affect, for example, non-structural proteins such as NSP6, potentially counteracting the host’s innate immune response. However, the functional effects of these mutations remain to be fully characterized^29,72–74^.

### Recombination

Recombination is the exchange of genetic material between genomes, for example, of different SARS-CoV-2 variants. This process is crucial to the evolution of the virus as it can increase genetic variation and thus lead to novel selection advantages. Recombination events occur quite frequently in betacoronaviruses^75–79^. A prerequisite is a simultaneous infection event of a host cell with two different viral variants. During replication, portions of the parental genetic material of the two variants combine with each other so that the progeny viruses carry hybrid genomes. Betacoronavirus recombinants may differ in phenotype from their parental lineages and may outperform them in terms of replicative fitness^80^. Therefore, detection of recombinant variants requires close monitoring in SARS-CoV-2 genomic surveillance. At the same time, it also poses technical challenges: samples with a true recombinant virus must be distinguished from patient material containing coinfecting viruses that have not recombined, from contaminations, and from sequencing or genome reconstruction errors^81^.

Because recombination requires coinfection of the same cell with two genetically distinct viruses, it is most likely to occur when multiple viral lineages co-circulate and when viral prevalence is high^78^. Given the ongoing genetic diversification of SARS-CoV-2 and its wide transmission in the population, the discovery of recombinant viruses has become increasingly common^79,82^. This is consistent with the co-circulation of different viral lineages (e.g., leading to recombination between Delta and Omicron, or different Omicron-BA.2 sublineages) and the intensity of genomic surveillance that enables such detections. Pangolin lineages starting with the letter “X” have been established for several recombinants and their offspring, for example, **XD** (Delta x Omicron-BA.1), **XE** (BA.1 x Omicron-BA.2), **XF** (Delta x Omicron-BA.1), and **XBB** (Omicron-BJ.1 x Omicron-BA.2.75*). However, in the Pangolin system each letter combination is limited to three virus generations, after which a new letter combination is assigned to subsequent descendant lineages^32^. Because letter selection is random and based on availability, tracing the relationships between lineages and sublineages can be complicated.

## Chronology of variant evolution

### Low diversity and one adaptive change

The first mutation with a clear impact on the epidemiology of SARS-CoV-2 was an amino acid substitution in the spike protein (S) [S:D614G; i.e., aspartic acid (D) in position 614 is substituted by glycine (G)]. Viral variants harboring the D614G substitution were rare at the onset of the pandemic but expanded rapidly in early 2020, becoming globally dominant by March 2020^58^ (Fig. 2A and Supplementary Figs. S1, S2). It was initially debated whether this sharp increase was due to a fitness advantage of 614 G variants, leading to their natural selection, or to a founder effect, where 614 G variants initiated, by sheer chance, most transmissions in multiple locations^44,83^. Subsequent comprehensive studies demonstrated an intrinsic transmission advantage, i.e., that the D614G substitution represented indeed an adaptive change that was naturally selected (see Box 1 for details and references). In January 2025, S:D614G was present in 99.2% of sequenced viruses in the international GISAID data.

### Rise of the VOCs

Apart from the expansion of D614G, little sequence diversity and evolution were observed during the first year of the pandemic (Fig. 3). This was partly due to limited sequencing efforts, resulting in undersampling (Supplementary Fig. S3), but also because at that time a broad range of public health measures was in place to prevent the spread of SARS-CoV-2 through unvaccinated populations worldwide, resulting in overall low case numbers^40,44,84–86^. However, as cases rose and genomic surveillance efforts were increased at the end of 2021, several virus variants displaying high sequence divergence and clear signals of an epidemiologic growth advantage emerged. The WHO coined the term Variant of Concern (VOC) in 2020, then defined as a virus variant with altered phenotype characteristics demonstrated to adversely affect epidemiology (especially transmissibility), clinical presentation (especially pathogenicity), or the effectiveness of countermeasures such as diagnostics, vaccines, or therapeutics^87^.

The first VOCs to emerge were B.1.1.7 (later designated as Alpha), B.1.351 (Beta), and P.1 (Gamma). These variants were initially termed 501Y.V1, 501Y.V2, and 501Y.V3 because they shared the S:N501Y substitution, which increases affinity to the ACE-2 receptor, thereby enhancing viral fitness^88–91^. Alpha displayed intrinsic higher transmissibility, Beta and Gamma were immune evasive. The spread of Beta and Gamma remained largely constrained to world regions that had seen high transmission levels during the first year of the pandemic^92–94^. There, the ability to evade the broad population immunity established through rampant infections turned out to be an evolutionary advantage for SARS-CoV-2, opening the evolutionary niche of reinfection^44^. In many other world regions, Alpha, with a reproduction number exceeding that of the index virus by 50-100%^95^, quickly became the predominant variant, driving a major surge of infections, severe illness, and death^96–100^.

In 2021, Alpha was displaced by Delta, the fourth variant declared a VOC, which emerged in India. With an R0 of 6-7^101^, Delta was intrinsically more transmissible than Alpha. The S:L452R, T478K, and P681R substitutions, which influence the affinity and cleavability of the spike, contributed to Delta’s increased transmissibility (see Box 2). In addition, this VOC was moderately immune evasive, transmitting efficiently through hosts that had been previously infected. Delta’s higher replication rates and more pronounced capacity to induce syncytium formation in airway cells translated to greater severity, increasing hospitalization and ICU admission rates. Delta became the dominant variant worldwide in June 2021 (Fig. 2A). Notably, the national vaccination campaigns in some countries, such as Germany, contributed substantially to decelerating the nascent Delta wave emerging in the summer of 2021^59^.

### Emergence and dominance of Omicron marked a watershed moment in the pandemic

At the end of 2021, the emergence of a new lineage prompted a swift, global response, thereby demonstrating the effectiveness of the genomic surveillance systems that had been implemented during the pandemic. The variant B.1.1.529, subsequently designated as Omicron, rapidly increased in the South African genome surveillance, a piece of information immediately shared with public health agencies worldwide^67^. Due to the new variant’s concerning constellation of mutations and the pronounced growth (transmission) advantage observed in a country with a high level of population immunity, it was quickly declared as a new VOC^102^, prompting increased surveillance measures and risk assessment studies worldwide. This facilitated the rapid accumulation and synthesis of epidemiologic, clinical, and laboratory experimental data that enabled a comprehensive assessment of the growth advantage of Omicron and the mechanisms underpinning it. Neutralization assays showed that the immune evasion displayed by Omicron was profound (albeit not complete), and its magnitude resembled an antigenic shift in the risk assessment framework of influenza viruses^103–107^. Consistent with these wet-lab data, integrated genomic and epidemiologic surveillance data demonstrated a propensity for infection of immunologically experienced individuals who had either been vaccinated or previously infected^108,109^. Moreover, high-validity tissue culture models indicated that Omicron had altered its host cell tropism to infect cells in the upper respiratory tract preferentially, enabling intrinsically more efficient person-to-person transmission^63,110^. Over the next months, it became gradually apparent that clinical, epidemiological, and genomic data lined up with these ex vivo data, at least for immunologically healthy and vaccinated adults. Omicron infections were associated with lower severity than infections with the previous VOCs. Although case counts reached record levels due to the numerous transmissions, ICU admissions and deaths did not exceed the levels that many countries experienced during the preceding Delta waves. While efficient suppression of Omicron transmissions is possible and has been demonstrated, e.g., in health care settings^111,112^, the reports on milder illness, the increasing vaccination rates, the overall pandemic fatigue, and the perception that containment of Omicron was not feasible led several countries to ease public health restrictions, often at the peak of the first Omicron wave. Healthcare systems in those countries were strained severely but were not overwhelmed and many countries transitioned to “living with COVID” mitigation policies. Thus, the emergence of Omicron may be viewed as a watershed moment. While mitigation policies may imply the absence of any transmission control, several measures aimed to curb the spread of COVID-19 were maintained (i.e., “vaccine plus” strategies), based on the long-term morbidity associated with COVID-19, the unknown impact of repeated SARS-CoV-2 infections, and the fact that elderly and immunologically compromised individuals remain vulnerable to severe acute illness.

### The Omicron complex

Omicron became globally dominant (frequency > 50% on GISAID) in January 2022 and has since then effectively displaced all other lineages. BA.2 was the first Omicron sublineage to predominate worldwide. BA.4 and BA.5 have identical S-Proteins that, relative to BA.2, contain three amino acid changes (L452R, F486V, and R493Q) and one deletion (del 69-70). L452R, F486V, and del 69-70 enhance infectivity while L452R and F486V increase resistance to neutralizing antibodies. R493Q, a reversion, is thought to restore receptor affinity which is compromised by F486V in some experimental models. The combination of these mutations led to an effective transmission advantage for BA.5, which became globally dominant in the 2022 summer^113–115^.

The BA.5 wave was succeeded by a “variant soup” of co-circulating Omicron sublineages, with regional differences in prevalence. These sublineages convergently acquired mutations at key residues in the spike receptor-binding domain (RBD), including R346, L452, N460, and F486. Mutations like L452R and F486S enhanced immune evasion, including resistance to monoclonal antibodies such as Bebtelovimab and Tixagevimab, while R346T and N460K improved ACE2 binding and infectivity^113,115–119^. Thus, by altering the antigenicity and ACE2 affinity of the spike, these adaptations increased the effective fitness (R_ᴇ_) of Omicron sublineages. Among them, BQ.1.1 and XBB emerged as the fittest, co-circulating globally until the end of 2022, when BQ.1.1 was outcompeted by XBB.1.5. Compared to its parental XBB lineage, XBB.1.5 carries the F486P substitution, which confers similar immune evasion and increased ACE2 affinity^82,115,120^. Further information on Omicron sublineages that emerged later is provided in Supplementary Table S1.

Over 56% of SARS-CoV-2 lineages and sublineages with sequence data available on GISAID (accessed January 29, 2025) descend from the parental Omicron lineages BA.1, BA.2, BA.3, BA.4, and BA.5 (including their sublineages), constituting the Omicron complex. Lineages within the Omicron complex continue to genetically diversify in an incremental adaptive process marked by convergent evolution of advantageous mutations. Many of these mutations are located on the spike and enable escape from the humoral immunity induced by previous variants, including earlier Omicron variants^70^. This contrasts the “saltatory” evolution observed with the historic emergence of VOCs. On the other hand, SARS-CoV-2 is known to persistently replicate in individuals with chronic infection^46,50,121,122^. Moreover, Delta and other de-escalated VOCs continue to circulate in animal reservoirs^123^. Thus, highly divergent lineages may continue to evolve but cannot be immediately detected via genomic surveillance for some time. A conceivable scenario is that one such lineage acquires characteristics that enhance human-to-human transmission, giving rise to a new VOC.

## Genomic surveillance in the context of public health activities

Genomic surveillance has played an essential role in tracking SARS-CoV-2 since the onset of the pandemic and enabled a deeper understanding of the virus’s spread and evolution, thus has been fundamental in shaping effective public health measures and interventions^43,59,67,92,93,95,124–128^. Over the pandemic, many countries implemented extensive testing and sequencing programs that facilitated the identification of cases and a high-resolution genomic surveillance both temporally and spatially. However, after WHO declared that COVID-19 is an established and ongoing health issue which no longer constitutes a public health emergency of international concern (PHEIC) on 5^th^ May 2023^129^, these activities have seen a noticeable decline. Medium-scale genomic surveillance systems often operate at a lower resolution, while effectively capturing essential aspects of the viral population dynamics and infection patterns. This can result in delayed detection of virus variants with unique phenotypes, primarily due to the limitations imposed by smaller sample sizes. Thus, the future intensity of SARS-CoV-2 genomic surveillance will be modified to a less comprehensive but flexible set up, aiming to balance the needs and costs of genomic surveillance against the burden of the disease. It is important to note and consider that the virus continues to evolve in a population with widespread immunity, often without causing severe illness^130^. However, accurately assessing the disease burden is challenging due to the evolution of SARS-CoV-2 and the factors influencing its spread^44,62^. The most effective surveillance integrates epidemiological and clinical patient data with virus’ genetic information in a timely manner. The genomic surveillance system still in place in Germany, operational since 2020, utilizes a nationwide network of laboratories (IMSSC2) and centralized sequencing at Robert Koch Institute; virus genomic data is to be complemented with clinical-epidemiological data provided by local health authorities^59^. In addition, SARS-CoV-2 is being integrated within the existing surveillance systems for acute respiratory infections with high public health impact (Influenza, RSV): Syndromic and virological surveillance is conducted through Germany’s national sentinel system, which covers both Severe Acute Respiratory Infection (SARI) and ambulatory Acute Respiratory Infections (ARI), providing a comprehensive view of acute respiratory illnesses^85,131–133^. These crucial surveillance instruments provide opportunities to correlate clinical symptoms or severity with different virus types and to identify and potentially isolate viruses with unusual phenotypic characteristics^134^. Thus, they are key in identifying and characterizing virus variants with distinctive traits, such as pronounced immune evasion, increased virulence, or reduced vaccine effectiveness. Facilitating prompt and focused research into the virulence and public health implications of newly emerging variants using these tools will be pivotal in guiding data-driven public health strategies. This includes making informed decisions about vaccine formulations and other intervention measures to effectively combat emerging viral threats and where such surveillance systems can make a strong contribution. These surveillance tools typically focus on a representative selection of symptomatic individuals. In addition, the COVID-19 pandemic has provided an opportunity to enhance these well-established monitoring systems with wastewater surveillance. Despite several experimental and bioinformatic challenges, wastewater surveillance is considered an overall promising complementary surveillance tool, as it provides virus information on asymptomatic infections and may enable early trend detection and population-scale monitoring^135^. However, this instrument does usually not allow for virus isolation or assessment of viral phenotypes. As a complementary system, wastewater surveillance represents a broad approach to track viral prevalence and trends across communities.

## Variant risk assessment by integration of virological, clinical-epidemiological, and genomic data

For public health purposes, assessing the risk posed by a new variant is of the utmost importance.

Whenever a new variant shows evidence of faster spread, i.e., an epidemiological growth advantage, this may be due to viral characteristics that favor transmission, i.e., a true transmission advantage or chance^136^. The public health risk emanating from the variant requires careful evaluation, the extent of which depends on the certainty of the observed growth advantage^36^. In the first stage, risk assessment involves confirmation of faster spread in different geographic regions as well as bioinformatic analysis, especially of the spike region, for mutations that might affect transmission efficiency^61^. Sequence analyses should be complemented by laboratory experimental characterization of the new variant’s virological phenotype. These experiments, performed in selected in vitro and in vivo models, assess the new variant’s growth characteristics, immunoevasive properties, and pathogenicity (virulence) under tightly controlled conditions. In addition, high-throughput assays have been developed to define more precisely epitopes of neutralizing antibodies and quantify the phenotypic effects of virtually any mutation of the spike protein, including those not yet observed in circulating variants^137,138^. Another essential part of variant risk assessment are epidemiological analyses to assess the clinical severity of the associated illness, which, combined with the growth advantage estimate, help to evaluate the risk of healthcare systems being strained or even overwhelmed by the new variant. Risk assessment analyses are most accurate when they are based on integrated clinical and genomic data^126,127,139,140^.

## Clinical presentation

### Acute illness

Symptomatic acute SARS-CoV-2- infection manifests with nonspecific symptoms after an incubation period of 4.2 days on average for the Omicron variant which is shorter than for infections with the Delta variant^141,142^. While the phase of active virus replication and shedding in the respiratory tract is very short in most patients, severely immunocompromised patients may exhibit prolonged viral persistence^143,144^.

The clinical presentation varies widely, ranging from asymptomatic or mild upper respiratory symptoms (predominantly) to interstitial pneumonia, which may take a severe clinical course and be complicated by Acute Respiratory Distress Syndrome (ARDS)^145^. Symptoms of a mild SARS-CoV-2 infection are non-specific (e.g. headache, fever and myalgia, sore throat, rhinitis) and are similar to other respiratory viral infections^146,147^. However, the leading symptoms appear to be somewhat related to the virus variant. For example, a sore throat is more likely observed in Omicron infection than in Delta infection^141^. A relatively specific COVID-19 symptom is an impairment of smell or taste^148,149^. However, depending on the study, there are often contradictory data about the association of this symptom with virus variants. While some studies report that changes in sense of smell and taste occurred more frequently with the Delta variant than with the Alpha variant, other studies suggest that this was a more typical symptom of the Alpha variant^150–152^. It seems that in addition to the increase in the frequency of sore throats, a decrease in smell or taste impairment is also typical in those infected with Omicron^150,153^. The correlation of headaches also appeared to decrease in studies with the evolution of the virus and was no longer significant with Omicron^154–156^. The proportion of asymptomatic infections and of non-severe COVID-19 cases is also reported to be significantly higher for Omicron than for Delta^157^; in addition to lower intrinsic virulence of Omicron, this trend may also be influenced by the generally higher population immunity present during the Omicron era.

However, all the aforementioned differences reported in some studies do not allow a reliable diagnosis of COVID-19 or even differentiation of virus variants based on the symptom constellation. The only way to identify a case with certainty is to test for specific antigens or RNA. To date, all variants have been detectable with the established diagnostic assays.

Severe COVID-19 manifests initially by cough and hypoxaemia due to interstitial pneumonia^158^ and may result in sepsis-like symptoms and organ failure. This is consistent with the widespread expression of the ACE2 receptor in numerous human tissues. SARS-CoV-2 can infect cells in various organ systems beyond the respiratory tract, resulting in a broad spectrum of sometimes severe extrapulmonary manifestations and complications^159–162^. Underlying pathomechanisms include: (i) cytolysis, i.e., direct damage to host cells by the replicating virus, (ii) a dysregulated, exuberant immune response that can lead to a life-threatening cytokine storm^163^, (iii) organ-specific inflammatory responses^159,164–167^, and (iv) endothelial damage which may be associated with dysregulation of the renin-angiotensin system and may cause, e.g., thrombo-embolic complications^168,169^. In addition to thromboembolic complications, other cardiovascular manifestations such as myocarditis, arrhythmias or myocardial infarction can also occur as a result of SARS-CoV-2 infection^170–172^. Acute kidney failure and several neurological manifestations such as stroke are also observed^173–177^. The involvement of other organ systems can lead to a complete picture of multi-organ failure and determine the outcome^178,179^.

During the initial phase of the pandemic, severe courses of COVID-19 were observed in a significant number of cases, even among young and previously healthy individuals, providing a rationale for implementing society-level measures to curb the spread of SARS-CoV-2. A meta-analysis showed that infections with the Delta variant had the highest severity. However, the hospitalization rate (but not the need for intensive care nor fatality rate) was higher for the Beta variant^180^.Currently, severe illness primarily affects individuals who are immunocompromised or have other predisposing conditions that put them at risk for severe illness, including advanced age^181^. This shift can be attributed to at least two factors: (i) most individuals have acquired immunity against the virus, protecting them not necessarily against asymptomatic or symptomatic infection but against severe clinical course^182–184^, and (ii) practically all infections are meanwhile caused by Omicron variants, generally considered of lower intrinsic pathogenicity than previously circulating variants^27,110,185–187^, even though this has been controversially discussed^188–191^. Thus, although widespread vaccination has successfully reduced the burden of severe COVID-19 disease in the general population, additional efforts will continuously be needed to protect the vulnerable.

A rare clinical manifestation observed in children following COVID-19 is Pediatric Inflammatory Multisystem Syndrome (PIMS), also known as Multisystem Inflammatory Syndrome in Children (MIS-C). This post-acute complication is associated with a dysregulated immune response after acute SARS-CoV-2-infection. It is hypothesized that this condition may be due to viral persistence leading to excessive activation of T-cells^192,193^. More recent data suggests that during the Omicron wave, both the frequency and severity of MIS-C have decreased, which may be due to the properties of Omicron or a potential protective effect of vaccination^194–197^.

### Late complications

#### Long COVID and post-COVID condition

Most COVID-19 patients recover within several days to weeks after infection. However, a significant number of individuals report various persistent or new physical or neurocognitive symptoms, even after an initial recovery from the acute SARS-CoV-2-infection. These include fatigue, exercise intolerance, malaise, dyspnea, orthostatic dysregulation, and neurocognitive dysfunction^198–201^. These sequelae can be prolonged, experienced as severely debilitating, and negatively impact daily functioning and quality of life. If such symptoms persist or recur and cannot be otherwise explained, they are referred to as Long COVID (beginning four weeks after acute infection) and post-COVID-19 condition (beginning 12 weeks after acute infection)^202^.

During or after COVID-19, neurocognitive symptoms may develop, including “*brain fog*”, memory loss, impaired consciousness, and confusion (so-called “neuro-COVID”)^203–206^. Whether these clinical sequelae are related to the radiographically observed structural brain changes observed after COVID-19^207–209^ has not yet been conclusively determined.

The available data may indicate that infection with the Omicron variant leads to fewer long-lasting COVID symptoms compared to earlier variants. Several hypotheses have been proposed to explain the decline in the incidence of long-term effects of COVID-19 in subsequent SARS-CoV-2 variants, including the increase in pre-existing immunity in the population over the course of the pandemic or changes in the pathogenicity of the virus^210,211^. The influence of reinfection as well as the vaccination status and timing must also be considered when interpreting the data^210,211^. However, the risk of post-COVID syndrome remains significant even in vaccinated individuals who have contracted SARS-CoV-2 in the Omicron era. Fatigue appears to be the most common post-COVID-19 symptom regardless of the SARS-CoV-2 variant^212^.

#### Other long-term sequelae

In addition, there is evidence that organ-specific complications^213^ and new-onset chronic non-communicable diseases^214^ may occur as long-term consequences of SARS-CoV-2 infection, even in individuals who were vaccinated and/or did not experience severe illness^214–217^. Emerging data suggest an increased risk for cardiovascular events, such as stroke, heart attack, arrhythmia, myocarditis, and heart failure^218^, as well as diabetes^219,220^, renal failure^221^, and psychiatric disorders^222,223^. The risk of postacute COVID-19 sequelae has been shown to be substantial even among vaccinated individuals who infected during the period of Omicron predominance^211^.

### Relationship of viral mutations to clinical presentation and severity

Pathogen genetic variation can impact virulence, which is an important determinant of clinical severity. Due to the significant influence of many other factors, including host immunity / vaccination status, age, and individual predisposition including the vascular system, it is challenging to assess pathogen virulence (or changes thereof) based on clinical signs and severity even when disease severity may be compared for SARS-CoV-2 variants co-circulating in a given population during the same time period^72^. It is similarly difficult, if not more so, to assess the impact of specific mutations on SARS-CoV-2 virulence based on severity data alone. Nevertheless, several amino acid substitutions that may affect acute disease severity have been identified based on clinical-epidemiological evidence.

A significant correlation with a higher viral load was found for the substitutions R203K and G204R in the N protein and S:D614G. However, although higher viral load is considered predictive of morbidity and mortality^224,225^, a statistically significant association could not be proven for these mutations^226^. A 382-nucleotide deletion that truncates open reading frame 7b (ORF7b) and eliminates ORF8 correlates with reduced clinical severity^227^. Notably, any ORF8 knockout appears to be associated with milder illness^228^. The P25L substitution in ORF3a has the potential to contribute to immune evasion and enhanced virulence, and has been linked to higher case fatality rates^226^. For the nt14408 mutation in *RdRp*, a higher single-nucleotide variant frequency was observed in severe cases^226^. The 11,083 G > U mutation has been associated with asymptomatic cases^226,227^. For mutations in ORF1ab and in the N gene, an association with asymptomatic outcomes has been described, found to be particularly strong for the co-occurring ORF1ab substitutions R6997P and V30L^226^. For mutations in NSP6, as well as other nonstructural proteins, an association with adverse clinical outcomes has been described^229^.

The differences between the variants in terms of acute clinical presentation and severity have prompted numerous investigations using in vitro and animal models to elucidate the role of specific mutations. The severe disease outcomes of the Delta variant are attributed to its enhanced replicative capacity and increased syncytium formation (fusogenicity) in the lung. Key mutations driving these features have been identified in the spike protein and include the L452R and P681R substitutions^230^ (see Box 2). In contrast, the reduced pathogenicity of Omicron variants is explained by their propensity for upper (rather than lower) respiratory tract replication and diminished syncytium formation; these features have been linked primarily to the Omicron spike. Mutations near the furin cleavage site (P681H, H655Y, and N679K) and the S1 C-terminus (T547K and H655Y) render the Omicron spike less fusogenic and less efficiently cleaved, suggesting it is a key determinant of the attenuated phenotype^28,110,231,232^. While most investigations have focused on spike protein mutations, a growing body of evidence suggests that non-spike mutations also impact SARS-CoV-2 virulence^226,227,229^, including animal experimental data indicating that mutations in NSP 14, envelope, and membrane proteins reduce the neuropathogenicity of Omicron^233^. NSP6 mutations modulate Omicron virulence^74,231^; and ORF8 mutations modulate lung inflammation as well as disease severity^231,234^.

With respect to the symptomatology of Long COVID, no single SARS-CoV-2 mutation has been definitively tied to specific Long COVID manifestations. However, some but not all studies indicate that variant-specific trends exist (reviewed in ref. ^235^): Omicron variants have been associated more frequently with gastrointestinal and musculoskeletal complaints and less frequently with cardiopulmonary and neuropsychiatric symptoms than pre-Omicron variants, although these findings are not entirely consistent in the literature^235–240^.

### Antiviral therapeutic options and resistance

Protease and polymerase inhibitors are employed as early direct antiviral therapy. Monoclonal antibodies, widely used for antiviral therapy during the first two years of the pandemic, currently have limited role in the treatment and prevention of COVID-19. This is because all viruses in circulation are Omicron variants, which are known for their significant immune evasion leading to decrease and even complete loss of the effectiveness of the licensed monoclonal antibodies (all of which were developed based on pre-Omicron variants)^104,241–245^. For pre-exposure prophylaxis, a new antibody has been approved, which is effective against XBB.1.5, XBB.1.16, XBB.2.3 and BA.2.86, but not against VOC carrying the F456L substitution^246^. In addition to antivirals, anti-inflammatory and immunomodulatory agents such as corticosteroids and IL-6 receptor antagonists, are available for the treatment of moderate to severe COVID-19 pneumonia requiring respiratory support.

Direct antiviral therapy inhibits viral replication and should be administered in the early phase of infection, also known as the “replicative phase”. Unlike monoclonal antibodies, which target the viral spike protein, these antivirals are not impacted by the presence of *Spike* gene mutations. However, antiviral resistance may evolve during treatment: Random transcription errors during viral RNA synthesis may result in inhibitor-specific point mutations in the therapeutic target proteins, which are selected if the virus replicates under continuous therapeutic pressure, thus giving rise to antiviral-resistant variants. Notably, a single amino acid substitution may suffice to render the virus less sensitive or resistant to an antiviral. As of today, four years after the first antiviral compounds were approved for COVID-19 therapy, resistance mutations are rare, occur mostly in laboratory settings, and have a worldwide prevalence of less than 1%^247–249^. Box 5 provides detailed information on the direct antiviral therapeutics currently approved for treating COVID-19.

BOX 5: Direct antiviral therapeutics Nirmatrelvir/ritonavir and Ensitrelvir. Nirmatrelvir inhibits the main protease of SARS-CoV-2 (Mpro), preventing it from cleaving the long polyprotein of the virus into shorter active proteins, an essential step in SARS-CoV-2 replication. To slow down the rapid degradation of nirmaltrevir by cytochrome P450 isoenzyme 3A4 (CYP3A4), it is combined with the protease inhibitor ritonavir^333^. The risk of inducing ritonavir resistance in people living with HIV during treatment with nirmatrelvir/ritonavir is considered minimal. For both nirmatrelvir and ensitrelvir, an MPro inhibitor licensed for COVID-19 treatment in Japan^334,335^, there is some indication that treatment during acute illness may reduce the risk of Long COVID syndrome^336–338^.

Remdesivir. Remdesivir is an RNA polymerase inhibitor with a structure similar to the RNA building block adenosine. It is a monophosphoramidate prodrug that is converted to the active metabolite remdesivir triphosphate in the target cells. Remdesivir has antiviral activity against RNA viruses, including coronaviruses such as SARS-CoV-2 and MERS-CoV, Ebola virus, and respiratory syncytial virus (RSV). It selectively inhibits viral RNA polymerase and, thus, viral RNA synthesis and viral replication. When remdesivir triphosphate is incorporated into the nascent viral RNA in place of adenosine triphosphate, the RNA chain is extended by three additional RNA building blocks before the copying process stops. Steric hindrance, caused by two atoms in the structure of the remdesivir, blocks the polymerase and stops viral replication by terminating RNA synthesis333. Remdesivir was the first antiviral to demonstrate clinical efficacy against COVID-19^339–341^. Molnupiravir. Molnupiravir is a nucleoside analogue prodrug that is rapidly metabolized by intracellular processes into N-hydroxy-cytidine triphosphate (NHC-TP). Incorporation of NHC-TP into viral RNA by the viral RNA-dependent RNA-polymerase leads to an accumulation of errors in the viral genome (“error catastrophe”) so that the virus no longer produces replicable progeny^342,343^. The clinical efficacy of molnupiravir has been controversially discussed^344–346^. Moreover, there are concerns that molnupiravir may induce transmissible, hypermutated SARS-CoV-2 strains^347^. After completing its evaluation, the European Medicines Agency (EMA) recommended to refuse marketing authorization.The manufacturer subsequently withdrew its application for European marketing authorization, meaning the drug is no longer available in EU member states^348^.

## Conclusions and perspectives

This article summarizes relevant data accumulated during the first four years (2020 – 2023) of the COVID-19 pandemic in the context of a “Working Group for Diagnostic and Evolution” on the causative agent, SARS-CoV-2, at Robert Koch-Institute, the German Public Health Institute. This has been done with the aim to underline the importance of virologic surveillance under the public health perspective and with respect to pandemic preparedness in future. The insights and data presented, while rooted in the German context, reflect broader virological and public health principles that are applicable across countries. The knowledge gained from these experiences can inform global strategies for surveillance and pandemic preparedness.

Thanks to substantial advances in sequencing of infective agents and the expansion of global data sharing initiatives, the scientific community has been able to detect and track the evolution of a newly introduced respiratory virus at unprecedented resolution and speed. However, it is still not possible to make reliable predictions about the long-term evolution of a new agent like SARS-CoV-2, which may continue to follow drift-like changes within the Omicron complex, but which could also be impacted by shift-like events, leading to new Variants of Concern (VOCs) that emerge for example from persistently infected humans or animal reservoirs. The genetic diversity of viruses, and hence also SARS-CoV-2, increases with the number of infections in both human and animal populations. This heightened genetic diversity benefits the virus, enabling rapid adaptation and the emergence of VOCs. Significant shifts resulting in VOCs have historically originated from chronically infected, immunocompromised human hosts^44,49,121,250,251^. Whether future SARS-CoV-2 VOCs will be derived primarily from Omicron or from phylogenetically divergent lineages, which may continue to evolve not only in animal reservoirs but also, potentially, in chronically infected humans, is currently uncertain^44,62^. The virulence of such future VOCs can also not be predicted^62^. Notably, it does not necessarily correlate with transmissibility, which is the virus’ key driver of positive selection.

As the virus continues to evolve, ongoing surveillance and research are crucial to understanding the impact of both spike and non-spike mutations on disease severity and clinical presentation. Research is ongoing to determine the impact of non-spike mutations on viral phenotype and pathogenicity. These mutations may affect viral replication, evasion of the innate immune response, and tissue tropism, potentially influencing disease outcomes. The specific mechanisms by which many of these mutations affect pathogenicity are still being investigated. Identification of key mutations linked to virologic properties will facilitate a more comprehensive understanding of the mechanisms of infection and aid in the development of antiviral treatments^252^.

Currently, SARS-CoV-2 circulation appears to trigger epidemiological surges at a higher frequency than that of influenza waves. This is likely due to rapid viral evolution and antigenic drift, indicating that SARS-CoV-2 has not yet evolved a clear pattern of seasonality. It is uncertain if and when the frequency of these waves will decrease in the future as population immunity becomes broader and more robust. It is similarly unclear whether or when the S protein of the virus will reach its evolutionary limits and constraints.

Therefore, it remains challenging to predict the evolutionary trajectory of SARS-CoV-2, including its potential for rapid adaptation and the emergence of new VOCs. Continuous in-depth surveillance that integrates genomic, clinical, epidemiologic, and virologic data obtained from various sources, including humans and animals as well as wastewater samples, enabling a fact-based risk assessment, is a public health imperative, which may inform timely interventions and the adaptation of vaccine formulation.

## Supplementary information

Supplementary Information

## Data Availability

DESH genome identifiers, intermediate data files, and scripts for visualizations are available at the Open Science Framework under https://osf.io/9r4ws. All used GISAID data are available through the GISAID identifier “EPI_SET_240415ce” and the corresponding 10.55876/gis8.240415ce. A corresponding Supplementary Table is provided (Supplementary Table S2).

## References

[1] Coronaviridae Study Group of the International Committee on Taxonomy of Viruses. The species Severe acute respiratory syndrome-related coronavirus: classifying 2019-nCoV and naming it SARS-CoV-2. *Nat. Microbiol.* **5**, 536–544 (2020). ***The Coronaviridae Study Group of the International Committee on Taxonomy of Viruses, a specialized panel of experts dedicated to the classification and nomenclature of coronaviruses designated the new coronavirus responsible for COVID-19 as SARS-CoV-2***. 10.1038/s41564-020-0695-z PMC7095448 32123347

[2] Graham, R. L. & Baric, R. S. Recombination, reservoirs, and the modular spike: mechanisms of coronavirus cross-species transmission. *J. Virol.* **84**, 3134–3146 (2010). 19906932 10.1128/JVI.01394-09 PMC2838128

[3] Fehr, A. R. & Perlman, S. Coronaviruses: an overview of their replication and pathogenesis. *Methods Mol. Biol.* **1282**, 1–23 (2015). 25720466 10.1007/978-1-4939-2438-7_1 PMC4369385

[4] Cui, J., Li, F. & Shi, Z. L. Origin and evolution of pathogenic coronaviruses. *Nat. Rev. Microbiol.* **17**, 181–192 (2019). 30531947 10.1038/s41579-018-0118-9 PMC7097006

[5] Laue, M. et al. Morphometry of SARS-CoV and SARS-CoV-2 particles in ultrathin plastic sections of infected Vero cell cultures. *Sci. Rep.* **11**, 3515 (2021). 33568700 10.1038/s41598-021-82852-7 PMC7876034

[6] Bai, C., Zhong, Q. & Gao, G. F. Overview of SARS-CoV-2 genome-encoded proteins. *Sci. China Life Sci.* **65**, 280–294 (2022). 34387838 10.1007/s11427-021-1964-4 PMC8362648

[7] Steiner, S. et al. SARS-CoV-2 biology and host interactions. *Nat. Rev. Microbiol.* **22**, 206–225 (2024). 38225365 10.1038/s41579-023-01003-z

[8] Khoury, D. S. et al. Neutralizing antibody levels are highly predictive of immune protection from symptomatic SARS-CoV-2 infection. *Nat. Med.* **27**, 1205–1211 (2021). ***First study demonstrating that in vitro neutralization levels are significantly correlated with immune protection against SARS-CoV-2, and thus allow to predict the efficacy of vaccines as they are being developed***. 10.1038/s41591-021-01377-8 34002089

[9] Enjuanes, L. et al. Development of protection against coronavirus induced diseases. A review. *Adv. Exp. Med. Biol.* **380**, 197–211 (1995). 8830481 10.1007/978-1-4615-1899-0_34

[10] Liu, L. et al. Potent neutralizing antibodies against multiple epitopes on SARS-CoV-2 spike. *Nature* **584**, 450–456 (2020). ***Landmark study reporting the isolation of multiple SARS-CoV-2-neutralizing monoclonal antibodies and revealing the epitopes of nineteen potent mAbs with potential for clinical development as therapeutic or prophylactic agents***. 32698192 10.1038/s41586-020-2571-7

[11] Baden, L. R. et al. Efficacy and safety of the mRNA-1273 SARS-CoV-2 vaccine. *N. Engl. J. Med.* **384**, 403–416 (2021). 33378609 10.1056/NEJMoa2035389 PMC7787219

[12] Polack, F. P. et al. Safety and efficacy of the BNT162b2 mRNA Covid-19 vaccine. *N. Engl. J. Med.* **383**, 2603–2615 (2020). 33301246 10.1056/NEJMoa2034577 PMC7745181

[13] Heath, P. T. et al. Safety and efficacy of NVX-CoV2373 Covid-19 vaccine. *N. Engl. J. Med.* **385**, 1172–1183 (2021). 34192426 10.1056/NEJMoa2107659 PMC8262625

[14] Hoffmann, M. et al. SARS-CoV-2 cell entry depends on ACE2 and TMPRSS2 and is blocked by a clinically proven protease inhibitor. *Cell* **181**, 271–280.e278 (2020). 32142651 10.1016/j.cell.2020.02.052 PMC7102627

[15] Jackson, C. B., Farzan, M., Chen, B. & Choe, H. Mechanisms of SARS-CoV-2 entry into cells. *Nat. Rev. Mol. Cell Biol.* **23**, 3–20 (2022). ***Review on the structural and cellular mechanisms of SARS-CoV-2 entry into host cells, focusing on the spike protein’s interactions with the ACE2 receptor, the roles of various proteases, and the implications for vaccines, antibodies, and other therapeutics***. 34611326 10.1038/s41580-021-00418-x PMC8491763

[16] Sungnak, W. et al. SARS-CoV-2 entry factors are highly expressed in nasal epithelial cells together with innate immune genes. *Nat. Med.* **26**, 681–687 (2020). 32327758 10.1038/s41591-020-0868-6 PMC8637938

[17] Zou, X. et al. Single-cell RNA-seq data analysis on the receptor ACE2 expression reveals the potential risk of different human organs vulnerable to 2019-nCoV infection. *Front. Med.* **14**, 185–192 (2020). 32170560 10.1007/s11684-020-0754-0 PMC7088738

[18] Hikmet, F. et al. The protein expression profile of ACE2 in human tissues. *Mol. Syst. Biol.* **16**, e9610 (2020). 32715618 10.15252/msb.20209610 PMC7383091

[19] Varga, Z. et al. Endothelial cell infection and endotheliitis in COVID-19. *Lancet* **395**, 1417–1418 (2020). 32325026 10.1016/S0140-6736(20)30937-5 PMC7172722

[20] Hamming, I. et al. Tissue distribution of ACE2 protein, the functional receptor for SARS coronavirus. A first step in understanding SARS pathogenesis. *J. Pathol.* **203**, 631–637 (2004). 15141377 10.1002/path.1570 PMC7167720

[21] Ziegler, C. G. K. et al. SARS-CoV-2 receptor ACE2 is an interferon-stimulated gene in human airway epithelial cells and is detected in specific cell subsets across tissues. *Cell* **181**, 1016–1035.e1019 (2020). 32413319 10.1016/j.cell.2020.04.035 PMC7252096

[22] Puelles, V. G. et al. Multiorgan and renal tropism of SARS-CoV-2. *N. Engl. J. Med.* **383**, 590–592 (2020). 32402155 10.1056/NEJMc2011400 PMC7240771

[23] Tavazzi, G. et al. Myocardial localization of coronavirus in COVID-19 cardiogenic shock. *Eur. J. Heart Fail* **22**, 911–915 (2020). 32275347 10.1002/ejhf.1828 PMC7262276

[24] Xiao, F. et al. Evidence for gastrointestinal infection of SARS-CoV-2. *Gastroenterology* **158**, 1831–1833.e1833 (2020). 32142773 10.1053/j.gastro.2020.02.055 PMC7130181

[25] Chertow, D. e. a. SARS-CoV-2 infection and persistence throughout the human body and brain. *Research Square*10.21203/rs.3.rs-1139035/v1 (2022).

[26] Stein, S. R. et al. SARS-CoV-2 infection and persistence in the human body and brain at autopsy. *Nature* **612**, 758–763 (2022). ***A study involving complete autopsies of 44 COVID-19 patients, revealing wide distribution of SARS-CoV-2 across the body, including the brain, with evidence of viral persistence up to 230 days post-symptom onset, despite minimal inflammation or direct viral damage outside the respiratory tract***. 36517603 10.1038/s41586-022-05542-y PMC9749650

[27] Meng, B. et al. Altered TMPRSS2 usage by SARS-CoV-2 Omicron impacts tropism and fusogenicity. *Nature*10.1038/s41586-022-04474-x (2022). 10.1038/s41586-022-04474-x PMC8942856 35104837

[28] Qu, P. et al. Determinants and mechanisms of the low fusogenicity and high dependence on endosomal entry of Omicron subvariants. *mBio* **14**, e0317622 (2023). 36625591 10.1128/mbio.03176-22 PMC9972997

[29] Kistler, K. E., Huddleston, J. & Bedford, T. Rapid and parallel adaptive mutations in spike S1 drive clade success in SARS-CoV-2. *Cell Host Microbe* **30**, 545–555.e544 (2022). ***This study revealed that the SARS-CoV-2 spike protein (in particular the S1 subunit) is the primary focus of rapid adaptive evolution, with a high mutation rate suggesting significant antigenic drift, and identified other important mutations, particularly in the Nsp6 protein***. 35364015 10.1016/j.chom.2022.03.018 PMC8938189

[30] Neher, R. A. Contributions of adaptation and purifying selection to SARS-CoV-2 evolution. *Virus Evol.* **8**, veac113 (2022). 37593203 10.1093/ve/veac113 PMC10431346

[31] Hadfield, J. et al. Nextstrain: real-time tracking of pathogen evolution. *Bioinformatics* **34**, 4121–4123 (2018). ***Nextstrain provides a comprehensive platform combining a viral genome database, bioinformatics tools, and interactive visualizations for real-time tracking of pathogen evolution and spread, integrating diverse data types for public health use: thus being of utmost importance for tracking SARS-CoV-2 evolution throughout the pandemic***. 29790939 10.1093/bioinformatics/bty407 PMC6247931

[32] Rambaut, A. et al. A dynamic nomenclature proposal for SARS-CoV-2 lineages to assist genomic epidemiology. *Nat. Microbiol.* **5**, 1403–1407 (2020). ***Introduction of the Pangolin nomenclature, a rational and dynamic naming system for SARS-CoV-2 that focuses on active and spreading lineages, which is being used by research groups and public health agencies worldwide***. 32669681 10.1038/s41564-020-0770-5 PMC7610519

[33] Harvey, W. T. et al. SARS-CoV-2 variants, spike mutations and immune escape. *Nat. Rev. Microbiol.* **19**, 409–424 (2021). 34075212 10.1038/s41579-021-00573-0 PMC8167834

[34] Alm, E. et al. Geographical and temporal distribution of SARS-CoV-2 clades in the WHO European Region, January to June 2020. *Euro Surveill.* **25**10.2807/1560-7917.ES.2020.25.32.2001410 (2020). 10.2807/1560-7917.ES.2020.25.32.2001410 PMC7427299 32794443

[35] World Health Organization. Tracking SARS-CoV-2 variants. https://www.who.int/en/activities/tracking-SARS-CoV-2-variants/ (2021).

[36] World Health Organization. Updated working definitions and primary actions for SARS-CoV-2 variants. https://www.who.int/publications/m/item/updated-working-definitions-and-primary-actions-for--sars-cov-2-variants (2023).

[37] Oh, D.-Y. et al. SARS-CoV-2-Varianten: Evolution im Zeitraffer. *Dtsch. Ärzteblatt* **118**, A-460/–B-388 (2021).

[38] World Health Organization. WHO Team Emergency Response (WRE), *COVID-19 Weekly Epidemiological Update* (2021).

[39] Lythgoe, K. A. et al. SARS-CoV-2 within-host diversity and transmission. *Science* **372**10.1126/science.abg0821 (2021). 10.1126/science.abg0821 PMC8128293 33688063

[40] van der Toorn, W. et al. An intra-host SARS-CoV-2 dynamics model to assess testing and quarantine strategies for incoming travelers, contact management, and de-isolation. *Patterns* **2**, 100262 (2021). 33899034 10.1016/j.patter.2021.100262 PMC8057735

[41] Baumgarte, S. et al. Investigation of a limited but explosive COVID-19 outbreak in a German secondary school. *Viruses* **14**10.3390/v14010087 (2022). 10.3390/v14010087 PMC8780098 35062291

[42] Scholl, M. et al. Bus riding as amplification mechanism for SARS-CoV-2 transmission, Germany, 2021(1). *Emerg. Infect. Dis.* **30**, 711–720 (2024). 38526123 10.3201/eid3004.231299 PMC10977817

[43] Gu, H. et al. Genomic epidemiology of SARS-CoV-2 under an elimination strategy in Hong Kong. *Nat. Commun.* **13**, 736 (2022). 35136039 10.1038/s41467-022-28420-7 PMC8825829

[44] Markov, P. V. et al. The evolution of SARS-CoV-2. *Nat. Rev. Microbiol.* **21**, 361–379 (2023). ***Comprehensive review of SARS-CoV-2 evolution with a focus on mechanisms generating genetic variation while also discussing future evolutionary scenarios.*** 37020110 10.1038/s41579-023-00878-2

[45] Choi, B. et al. Persistence and evolution of SARS-CoV-2 in an Immunocompromised Host. *N. Engl. J. Med.* **383**, 2291–2293 (2020). 33176080 10.1056/NEJMc2031364 PMC7673303

[46] Chaguza, C. et al. Accelerated SARS-CoV-2 intrahost evolution leading to distinct genotypes during chronic infection. *Cell Rep. Med.* **4**, 100943 (2023). 36791724 10.1016/j.xcrm.2023.100943 PMC9906997

[47] Gonzalez-Reiche, A. S. et al. Sequential intrahost evolution and onward transmission of SARS-CoV-2 variants. *Nat. Commun.* **14**, 3235 (2023). 37270625 10.1038/s41467-023-38867-x PMC10239218

[48] Lustig, G. et al. SARS-CoV-2 infection in immunosuppression evolves sub-lineages which independently accumulate neutralization escape mutations. *Virus Evol.* **10**, vead075 (2024). 38361824 10.1093/ve/vead075 PMC10868398

[49] Ghafari, M., Liu, Q., Dhillon, A., Katzourakis, A. & Weissman, D. B. Investigating the evolutionary origins of the first three SARS-CoV-2 variants of concern. *Front. Virol.* **2**10.3389/fviro.2022.942555 (2022).

[50] Harari, S. et al. Drivers of adaptive evolution during chronic SARS-CoV-2 infections. *Nat. Med.* **28**, 1501–1508 (2022). 35725921 10.1038/s41591-022-01882-4 PMC9307477

[51] Marques, A. D. et al. SARS-CoV-2 evolution during prolonged infection in immunocompromised patients. *mBio* **15**, e0011024 (2024). 38364100 10.1128/mbio.00110-24 PMC10936176

[52] Ghafari, M. et al. Prevalence of persistent SARS-CoV-2 in a large community surveillance study. *Nature* **626**, 1094–1101 (2024). 38383783 10.1038/s41586-024-07029-4 PMC10901734

[53] Machkovech, H. M. et al. Persistent SARS-CoV-2 infection: significance and implications. *Lancet Infect. Dis*. 10.1016/S1473-3099(23)00815-0 (2024). ***Review on persistent SARS-CoV-2 infections, which highlights that their potential of going unrecognized, of contributing to tissue reservoirs thereby requiring new diagnostic and therapeutic strategies, and of generating new virus variants that can evade immunity poses significant clinical and public health challenges.***

[54] Khare, S. et al. GISAID’s role in pandemic response. *China CDC Wkly.* **3**, 1049–1051 (2021). 34934514 10.46234/ccdcw2021.255 PMC8668406

[55] Shu, Y. & McCauley, J. GISAID: global initiative on sharing all influenza data—from vision to reality. *Euro Surveill.* **22**10.2807/1560-7917.ES.2017.22.13.30494 (2017). 10.2807/1560-7917.ES.2017.22.13.30494 PMC5388101 28382917

[56] Elbe, S. & Buckland-Merrett, G. Data, disease and diplomacy: GISAID’s innovative contribution to global health. *Glob. Chall.* **1**, 33–46 (2017). 31565258 10.1002/gch2.1018 PMC6607375

[57] Focosi, D., Maggi, F., McConnell, S. & Casadevall, A. Very low levels of remdesivir resistance in SARS-COV-2 genomes after 18 months of massive usage during the COVID19 pandemic: a GISAID exploratory analysis. *Antivir. Res.* **198**, 105247 (2022). 35033572 10.1016/j.antiviral.2022.105247 PMC8755559

[58] Korber, B. et al. Tracking changes in SARS-CoV-2 spike: evidence that D614G increases infectivity of the COVID-19 virus. *Cell* **182**, 812–827.e819 (2020). ***This paper noted for the first time that the SARS-CoV-2 variant with the Spike protein change D614G became globally dominant, likely due to a fitness advantage; it underscored the need for ongoing genomic surveillance of SARS-CoV-2***. 32697968 10.1016/j.cell.2020.06.043 PMC7332439

[59] Oh, D. Y. et al. Advancing precision vaccinology by molecular and genomic surveillance of severe acute respiratory syndrome coronavirus 2 in Germany, 2021. *Clin. Infect. Dis.* **75**, S110–S120 (2022). 35749674 10.1093/cid/ciac399 PMC9278222

[60] Robert Koch-Institut. SARS-CoV-2 Sequenzdaten aus Deutschland (2023-06-16). *Zenodo*10.5281/zenodo.8046538 (2023).

[61] Raharinirina, N. A. et al. SARS-CoV-2 evolution on a dynamic immune landscape. *Nature*10.1038/s41586-024-08477-8 (2025). ***This paper presents a mechanistic model predicting SARS-CoV-2 variant dynamics based on the virus’s evolution to evade antibody-mediated neutralization in a population with varied immune histories due to global inequalities in vaccine distribution and infection-prevention measures.*** 10.1038/s41586-024-08477-8 PMC11882442 39880955

[62] Telenti, A., Hodcroft, E. B. & Robertson, D. L. The evolution and biology of SARS-CoV-2 variants. *Cold Spring Harb. Perspect. Med.*10.1101/cshperspect.a041390 (2022). 10.1101/cshperspect.a041390 PMC9159258 35444005

[63] Peacock, T. P. et al. The SARS-CoV-2 variant, Omicron, shows rapid replication in human primary nasal epithelial cultures and efficiently uses the endosomal route of entry. *bioRxiv*10.1101/2021.12.31.474653 (2022).

[64] Magnus, C. L. et al. Targeted escape of SARS-CoV-2 in vitro from monoclonal antibody S309, the precursor of sotrovimab. *Front. Immunol.* **13**, 966236 (2022). 36090991 10.3389/fimmu.2022.966236 PMC9449809

[65] Arruda, H. R. S. et al. Conformational stability of SARS-CoV-2 glycoprotein spike variants. *iScience* **26**, 105696 (2023). 36465857 10.1016/j.isci.2022.105696 PMC9710096

[66] Gobeil, S. M. et al. Effect of natural mutations of SARS-CoV-2 on spike structure, conformation, and antigenicity. *Science* **373**10.1126/science.abi6226 (2021). 10.1126/science.abi6226 PMC8611377 34168071

[67] Viana, R. et al. Rapid epidemic expansion of the SARS-CoV-2 Omicron variant in southern Africa. *Nature* **603**, 679–686 (2022). ***First description of the genomic profile and early transmission dynamics of the Omicron variant.*** 35042229 10.1038/s41586-022-04411-y PMC8942855

[68] Roemer, C. et al. SARS-CoV-2 evolution in the Omicron era. *Nat. Microbiol.* **8**, 1952–1959 (2023). 37845314 10.1038/s41564-023-01504-w

[69] Cao, Y. et al. Imprinted SARS-CoV-2 humoral immunity induces convergent Omicron RBD evolution. *Nature* **614**, 521–529 (2023). 36535326 10.1038/s41586-022-05644-7 PMC9931576

[70] Cao, Y. et al. BA.2.12.1, BA.4 and BA.5 escape antibodies elicited by Omicron infection. *Nature* **608**, 593–602 (2022). 35714668 10.1038/s41586-022-04980-y PMC9385493

[71] Ito, J. et al. Convergent evolution of SARS-CoV-2 Omicron subvariants leading to the emergence of BQ.1.1 variant. *Nat. Commun.* **14**, 2671 (2023). 37169744 10.1038/s41467-023-38188-z PMC10175283

[72] Carabelli, A. M. et al. SARS-CoV-2 variant biology: immune escape, transmission and fitness. *Nat. Rev. Microbiol.* **21**, 162–177 (2023). 36653446 10.1038/s41579-022-00841-7 PMC9847462

[73] Thorne, L. G. et al. Evolution of enhanced innate immune evasion by SARS-CoV-2. *Nature* **602**, 487–495 (2022). 34942634 10.1038/s41586-021-04352-y PMC8850198

[74] Bills, C. J. et al. Mutations in SARS-CoV-2 variant nsp6 enhance type-I interferon antagonism. *Emerg. Microbes Infect.* **12**, 2209208 (2023). 37114433 10.1080/22221751.2023.2209208 PMC10184609

[75] Bobay, L. M., O’Donnell, A. C. & Ochman, H. Recombination events are concentrated in the spike protein region of Betacoronaviruses. *PLoS Genet.* **16**, e1009272 (2020). 33332358 10.1371/journal.pgen.1009272 PMC7775116

[76] Corman, V. M. et al. Rooting the phylogenetic tree of middle East respiratory syndrome coronavirus by characterization of a conspecific virus from an African bat. *J. Virol.* **88**, 11297–11303 (2014). 25031349 10.1128/JVI.01498-14 PMC4178802

[77] Garvin, M. R. et al. Rapid expansion of SARS-CoV-2 variants of concern is a result of adaptive epistasis. *bioRxiv*10.1101/2021.08.03.454981 (2021).

[78] Jackson, B. et al. Generation and transmission of interlineage recombinants in the SARS-CoV-2 pandemic. *Cell* **184**, 5179–5188.e5178 (2021). 34499854 10.1016/j.cell.2021.08.014 PMC8367733

[79] Bolze, A. et al. Evidence for SARS-CoV-2 Delta and Omicron co-infections and recombination. *Med* **3**, 848–859.e844 (2022). 36332633 10.1016/j.medj.2022.10.002 PMC9581791

[80] Schroeder, S. et al. Functional comparison of MERS-coronavirus lineages reveals increased replicative fitness of the recombinant lineage 5. *Nat. Commun.* **12**, 5324 (2021). 34493730 10.1038/s41467-021-25519-1 PMC8423819

[81] Kreier, F. Deltacron: the story of the variant that wasn’t. *Nature* **602**, 19 (2022). 35058630 10.1038/d41586-022-00149-9

[82] Tamura, T. et al. Virological characteristics of the SARS-CoV-2 XBB variant derived from recombination of two Omicron subvariants. *Nat. Commun.* **14**, 2800 (2023). 37193706 10.1038/s41467-023-38435-3 PMC10187524

[83] Lauring, A. S. & Hodcroft, E. B. Genetic variants of SARS-CoV-2-what do they mean?. *JAMA* **325**, 529–531 (2021). 33404586 10.1001/jama.2020.27124

[84] Oh, D. Y., Bottcher, S., Kroger, S. & von Kleist, M. [SARS-CoV-2 transmission routes and implications for self- and non-self-protection]. *Bundesgesundheitsblatt Gesundheitsforschung Gesundheitsschutz* **64**, 1050–1057 (2021). 34324023 10.1007/s00103-021-03389-8 PMC8319698

[85] Oh, D. Y. et al. Trends in respiratory virus circulation following COVID-19-targeted nonpharmaceutical interventions in Germany, January - September 2020: analysis of national surveillance data. *Lancet Reg. Health Eur.* **6**, 100112 (2021). 34124707 10.1016/j.lanepe.2021.100112 PMC8183189

[86] von Kleist, M. et al. Abwägung der Dauer von Quarantäne und Isolierung bei COVID-19. *Epid. Bull.* **39**, 3–11 (2020).

[87] World Health Organization. COVID-19 Weekly Epidemiological Update (Suppl. 25 February 2021), Special Edition: Proposed working definitions of SARS-CoV-2 Variants of Interest and Variants of Concern. https://www.who.int/publications/m/item/covid-19-weekly-epidemiological-update (2021).

[88] Liu, Y. et al. The N501Y spike substitution enhances SARS-CoV-2 infection and transmission. *Nature* **602**, 294–299 (2022). 34818667 10.1038/s41586-021-04245-0 PMC8900207

[89] Prüß, B. M. Variants of SARS CoV-2: mutations, transmissibility, virulence, drug resistance, and antibody/vaccine sensitivity. *Front. Biosci.* **27**, 65 (2022). 10.31083/j.fbl2702065 35227008

[90] Starr, T. N. et al. Deep mutational scanning of SARS-CoV-2 receptor binding domain reveals constraints on folding and ACE2 binding. *Cell* **182**, 1295–1310.e1220 (2020). ***Comprehensive mutational analysis of the SARS-CoV-2 receptor binding domain (RBD) reveals that while most mutations reduce protein expression and ACE2 binding, some enhance ACE2 affinity, including at critical interface residues, offering insights for vaccine and therapeutic design, though these affinity-enhancing mutations have not been selected in current pandemic strains***. 32841599 10.1016/j.cell.2020.08.012 PMC7418704

[91] Zahradnik, J. et al. SARS-CoV-2 variant prediction and antiviral drug design are enabled by RBD in vitro evolution. *Nat. Microbiol.* **6**, 1188–1198 (2021). 34400835 10.1038/s41564-021-00954-4

[92] Tegally, H. et al. Detection of a SARS-CoV-2 variant of concern in South Africa. *Nature* **592**, 438–443 (2021). 33690265 10.1038/s41586-021-03402-9

[93] Faria, N. R. et al. Genomics and epidemiology of the P.1 SARS-CoV-2 lineage in Manaus, Brazil. *Science*10.1126/science.abh2644 (2021). 10.1126/science.abh2644 PMC8139423 33853970

[94] Sabino, E. C. et al. Resurgence of COVID-19 in Manaus, Brazil, despite high seroprevalence. *Lancet* **397**, 452–455 (2021). 33515491 10.1016/S0140-6736(21)00183-5 PMC7906746

[95] Volz, E. et al. Assessing transmissibility of SARS-CoV-2 lineage B.1.1.7 in England. *Nature* **593**, 266–269 (2021). 33767447 10.1038/s41586-021-03470-x

[96] European Centre for Disease Prevention and Control. Rapid increase of a SARS-CoV-2 variant with multiple spike protein mutations observed in the United Kingdom. ECDC Threat Assessment Brief (2020).

[97] Davies, N. G. et al. Increased mortality in community-tested cases of SARS-CoV-2 lineage B.1.1.7. *Nature* **593**, 270–274 (2021). ***Clinical-epidemiological analysis indicating that the Alpha variant is associated with a significantly higher risk of death, suggesting it may cause more severe illness***. 33723411 10.1038/s41586-021-03426-1 PMC9170116

[98] Challen, R. et al. Risk of mortality in patients infected with SARS-CoV-2 variant of concern 202012/1: matched cohort study. *BMJ* **372**, n579 (2021). 33687922 10.1136/bmj.n579 PMC7941603

[99] Paul, P. et al. Genomic surveillance for SARS-CoV-2 variants circulating in the United States, December 2020-May 2021. *Morb. Mortal. Wkly Rep.* **70**, 846–850 (2021). 10.15585/mmwr.mm7023a3 PMC8191868 34111060

[100] Dhanasekaran, V. et al. Air travel-related outbreak of multiple SARS-CoV-2 variants. *J. Travel Med.* **28**10.1093/jtm/taab149 (2021). 10.1093/jtm/taab149 PMC8522424 34542623

[101] Burki, T. K. Lifting of COVID-19 restrictions in the UK and the Delta variant. *Lancet Respir. Med.*10.1016/S2213-2600(21)00328-3 (2021). 10.1016/S2213-2600(21)00328-3 PMC8275031 34265238

[102] World Health Organization. *Classification of Omicron (B.1.1.529): SARS-CoV-2**Variant of Concern*. https://www.who.int/news/item/26-11-2021-classification-of-omicron-(b.1.1.529)-sars-cov-2-variant-of-concern (2021).

[103] Cele, S. et al. Omicron extensively but incompletely escapes Pfizer BNT162b2 neutralization. *Nature*10.1038/s41586-021-04387-1 (2021). 10.1038/s41586-021-04387-1 PMC8866126 35016196

[104] Liu, L. et al. Striking antibody evasion manifested by the Omicron variant of SARS-CoV-2. *Nature*10.1038/s41586-021-04388-0 (2021). 10.1038/s41586-021-04388-0 35016198

[105] Planas, D. et al. Considerable escape of SARS-CoV-2 Omicron to antibody neutralization. *Nature*10.1038/s41586-021-04389-z (2021). 10.1038/s41586-021-04389-z 35016199

[106] Schmidt, F. et al. Plasma neutralization properties of the SARS-CoV-2 Omicron variant. *N. Engl. J. Med.*10.1056/NEJMc2119641 (2021). 10.1056/NEJMc2119641 PMC8757565 35030645

[107] Rossler, A., Riepler, L., Bante, D., von Laer, D. & Kimpel, J. SARS-CoV-2 Omicron variant neutralization in serum from vaccinated and convalescent persons. *N. Engl. J. Med.* **386**, 698–700 (2022). 35021005 10.1056/NEJMc2119236 PMC8781314

[108] Collie, S., Champion, J., Moultrie, H., Bekker, L. G. & Gray, G. Effectiveness of BNT162b2 Vaccine against Omicron Variant in South Africa. *N. Engl. J. Med.*10.1056/NEJMc2119270 (2021). 10.1056/NEJMc2119270 PMC8757569 34965358

[109] Pulliam, J. R. C. et al. Increased risk of SARS-CoV-2 reinfection associated with emergence of Omicron in South Africa. *Science* **376**, eabn4947 (2022). 35289632 10.1126/science.abn4947 PMC8995029

[110] Hui, K. P. Y. et al. SARS-CoV-2 Omicron variant replication in human bronchus and lung ex vivo. *Nature* **603**, 715–720 (2022). 35104836 10.1038/s41586-022-04479-6

[111] Baker, M. A. et al. Rapid control of hospital-based severe acute respiratory syndrome coronavirus 2 Omicron clusters through daily testing and universal use of N95 respirators. *Clin. Infect. Dis.* **75**, e296–e299 (2022). 35137035 10.1093/cid/ciac113 PMC8903387

[112] Fuszl, A. et al. COVID-19 patient and personal safety—lessons learnt for pandemic preparedness and the way to the next normal. *Antimicrob. Resist. Infect. Control* **12**, 27 (2023). 37005696 10.1186/s13756-023-01231-1 PMC10066952

[113] Pastorio, C. et al. Impact of mutations defining SARS-CoV-2 Omicron subvariants BA.2.12.1 and BA.4/5 on Spike function and neutralization. *iScience* **26**, 108299 (2023). 38026181 10.1016/j.isci.2023.108299 PMC10661123

[114] Wang, Q. et al. Antibody evasion by SARS-CoV-2 Omicron subvariants BA.2.12.1, BA.4 and BA.5. *Nature* **608**, 603–608 (2022). 35790190 10.1038/s41586-022-05053-w PMC9385487

[115] Yajima, H. et al. Molecular and structural insights into SARS-CoV-2 evolution: from BA.2 to XBB subvariants. *mBio* **15**, e0322023 (2024). 39283095 10.1128/mbio.03220-23 PMC11481514

[116] Focosi, D., Quiroga, R., McConnell, S., Johnson, M. C. & Casadevall, A. Convergent evolution in SARS-CoV-2 spike creates a variant soup from which new COVID-19 waves emerge. *Int. J. Mol. Sci.* **24**10.3390/ijms24032264 (2023). 10.3390/ijms24032264 PMC9917121 36768588

[117] Qu, P. et al. Evasion of neutralizing antibody responses by the SARS-CoV-2 BA.2.75 variant. *Cell Host Microbe* **30**, 1518–1526.e1514 (2022). 36240764 10.1016/j.chom.2022.09.015 PMC9515334

[118] Wang, Q. et al. Antigenic characterization of the SARS-CoV-2 Omicron subvariant BA.2.75. *Cell Host Microbe* **30**, 1512–1517.e1514 (2022). 36108630 10.1016/j.chom.2022.09.002 PMC9444898

[119] Wang, Q. et al. Recurrent SARS-CoV-2 spike mutations confer growth advantages to select JN.1 sublineages. *Emerg. Microbes Infect.* **13**, 2402880 (2024). 39259045 10.1080/22221751.2024.2402880 PMC11407393

[120] Yamasoba, D. et al. Virological characteristics of the SARS-CoV-2 omicron XBB.1.16 variant. * Lancet Infect. Dis.* **23**, 655–656 (2023). 37148902 10.1016/S1473-3099(23)00278-5 PMC10156138

[121] Avanzato, V. A. et al. Case study: Prolonged infectious SARS-CoV-2 shedding from an asymptomatic immunocompromised cancer patient. *Cell*10.1016/j.cell.2020.10.049 (2020). 10.1016/j.cell.2020.10.049 PMC7640888 33248470

[122] Shafer, M. M. et al. Tracing the origin of SARS-CoV-2 omicron-like spike sequences detected in an urban sewershed: a targeted, longitudinal surveillance study of a cryptic wastewater lineage. *Lancet Microbe*10.1016/S2666-5247(23)00372-5 (2024). 10.1016/S2666-5247(23)00372-5 PMC11049544 38484748

[123] Caserta, L. C. et al. White-tailed deer (Odocoileus virginianus) may serve as a wildlife reservoir for nearly extinct SARS-CoV-2 variants of concern. *Proc. Natl. Acad. Sci. USA* **120**, e2215067120 (2023). 36719912 10.1073/pnas.2215067120 PMC9963525

[124] Davies, N. G. et al. Estimated transmissibility and impact of SARS-CoV-2 lineage B.1.1.7 in England. *Science*10.1126/science.abg3055 (2021). 10.1126/science.abg3055 PMC8128288 33658326

[125] Tegally, H. et al. Emergence of SARS-CoV-2 Omicron lineages BA.4 and BA.5 in South Africa. *Nat. Med.* **28**, 1785–1790 (2022). 35760080 10.1038/s41591-022-01911-2 PMC9499863

[126] Subissi, L. et al. An early warning system for emerging SARS-CoV-2 variants. *Nat. Med.* **28**, 1110–1115 (2022). ***Comment outlining the approaches used by WHO Technical Advisory Group on Virus Evolution (TAG-VE) to assess the need for public health action in response to emerging variants, calling for strengthening of the global seqeuncing and surveillance capacities in combination with multidisciplinary studies of infectivity, virulence and immune escape***. 35637337 10.1038/s41591-022-01836-w PMC11346314

[127] Subissi, L. et al. An updated framework for SARS-CoV-2 variants reflects the unpredictability of viral evolution. *Nat. Med*. 10.1038/s41591-024-02949-0 (2024). ***Presentation of the updated WHO framework for tracking and assessing SARS-CoV-2 variants, which could be adapted for other emerging respiratory diseases with epidemic and pandemic potential.*** 10.1038/s41591-024-02949-0 38720002

[128] Khan, K. et al. Evolution and neutralization escape of the SARS-CoV-2 BA.2.86 subvariant. *Nat. Commun.* **14**, 8078 (2023). 38057313 10.1038/s41467-023-43703-3 PMC10700484

[129] World Health Organization. Statement on the fifteenth meeting of the IHR (2005) Emergency Committee on the COVID-19 pandemic. https://www.who.int/news/item/05-05-2023-statement-on-the-fifteenth-meeting-of-the-international-health-regulations-(2005)-emergency-committee-regarding-the-coronavirus-disease-(covid-19)-pandemic (2023).

[130] Cohen, C. & Pulliam, J. COVID-19 infection, reinfection, and the transition to endemicity. *Lancet* **401**, 798–800 (2023). 36930672 10.1016/S0140-6736(22)02634-4 PMC9934854

[131] Biere, B., Oh, D. Y., Wolff, T. & Durrwald, R. Surveillance of endemic human Coronaviruses in Germany, 2019/2020. *Lancet Reg. Health Eur.* **11**, 100262 (2021). 34751265 10.1016/j.lanepe.2021.100262 PMC8566015

[132] Oh, D. Y. et al. Virological surveillance and molecular characterization of human parainfluenzavirus infection in children with acute respiratory illness: Germany, 2015-2019. *Microorganisms* **9**10.3390/microorganisms9071508 (2021). 10.3390/microorganisms9071508 PMC8307145 34361941

[133] Oh, D. Y. et al. Preparing for the next influenza season: monitoring the emergence and spread of antiviral resistance. *Infect. Drug Resist.* **16**, 949–959 (2023). 36814825 10.2147/IDR.S389263 PMC9939793

[134] World Health Organization. End-to-end integration of SARS-CoV-2 and influenza sentinel surveillance: compendium of country approaches. https://www.who.int/publications/i/item/9789240056701 (2023).

[135] Munteanu, V. et al. SARS-CoV-2 Wastewater Genomic Surveillance: Approaches, Challenges, and Opportunities. https://arxiv.org/abs/2309.13326 (2024). 10.1186/s13059-025-03927-6 PMC12794521 41526978

[136] Hodcroft, E. B. et al. Spread of a SARS-CoV-2 variant through Europe in the summer of 2020. *Nature* **595**, 707–712 (2021). 34098568 10.1038/s41586-021-03677-y

[137] Greaney, A. J. et al. Complete mapping of mutations to the SARS-CoV-2 spike receptor-binding domain that escape antibody recognition. *Cell Host Microbe* **29**, 44–57.e49 (2021). 33259788 10.1016/j.chom.2020.11.007 PMC7676316

[138] Greaney, A. J., Starr, T. N. & Bloom, J. D. An antibody-escape estimator for mutations to the SARS-CoV-2 receptor-binding domain. *Virus Evol.* **8**, veac021 (2022). 35573973 10.1093/ve/veac021 PMC9092643

[139] Salzberger, B. et al. An appeal for strengthening genomic pathogen surveillance to improve pandemic preparedness and infection prevention: the German perspective. *Infection* **51**, 805–811 (2023). 37129842 10.1007/s15010-023-02040-9 PMC10152431

[140] Worp, N. et al. Towards the development of a SARS-CoV-2 variant risk assessment tool: expert consultation on the assessment of scientific evidence on emerging variants. *Lancet Microbe* **4**, e830–e836 (2023). 37640039 10.1016/S2666-5247(23)00179-9

[141] Menni, C. et al. Symptom prevalence, duration, and risk of hospital admission in individuals infected with SARS-CoV-2 during periods of omicron and delta variant dominance: a prospective observational study from the ZOE COVID Study. *Lancet* **399**, 1618–1624 (2022). ***Large-scale observational study of the clinical features of COVID during periods of Omicron vs. Delta dominance, indicating that in vaccinated individuals Omicron is associated with different symptom patterns and milder illness***. 35397851 10.1016/S0140-6736(22)00327-0 PMC8989396

[142] Wu, Y. et al. Incubation period of COVID-19 caused by unique SARS-CoV-2 strains: a systematic review and meta-analysis. *JAMA Netw. Open* **5**, e2228008 (2022). 35994285 10.1001/jamanetworkopen.2022.28008 PMC9396366

[143] Kang, S. W. et al. Characteristics and risk factors of prolonged viable virus shedding in immunocompromised patients with COVID-19: a prospective cohort study. *J. Infect.* **86**, 412–414 (2023). 36682630 10.1016/j.jinf.2023.01.024 PMC9852259

[144] Zhang, X., Zhang, L., Zhang, K., Chen, Y. & Wang, L. Immunocompromised states caused the prolonged duration of viral shedding in middle-aged and elderly hemodialysis patients infected with the Omicron variant of COVID-19. *Ther. Apher. Dial.* **27**, 720–725 (2023). 36691341 10.1111/1744-9987.13969

[145] Long, B. et al. Clinical update on COVID-19 for the emergency clinician: presentation and evaluation. *Am. J. Emerg. Med.* **54**, 46–57 (2022). 35121478 10.1016/j.ajem.2022.01.028 PMC8779861

[146] Goller, K. V. et al. Clinical manifestations of infections with the Omicron sub-lineages BA.1, BA.2, and BA.5: a retrospective follow-up analysis of public health data from Mecklenburg-Western Pomerania, Germany. *Viruses* **16**10.3390/v16030454 (2024). 10.3390/v16030454 PMC10974208 38543819

[147] Wang, M. et al. Clinical characteristics of 1139 mild cases of the SARS-CoV-2 Omicron variant infected patients in Shanghai. *J. Med. Virol.* **95**, e28224 (2023). 36238984 10.1002/jmv.28224 PMC9874495

[148] Eliezer, M. et al. Loss of smell in patients with COVID-19: MRI data reveal a transient edema of the olfactory clefts. (Reader response by Vaira et al., 2021). *Neurology* **95**, e3145–e3152 (2020). 32917809 10.1212/WNL.0000000000010806

[149] Roland, L. T. et al. Smell and taste symptom-based predictive model for COVID-19 diagnosis. *Int. Forum Allergy Rhinol.* **10**, 832–838 (2020). 32363809 10.1002/alr.22602 PMC7267242

[150] Cojocaru, C., Cojocaru, E., Turcanu, A. M. & Zaharia, D. C. Clinical challenges of SARS-CoV-2 variants (Review). *Exp. Ther. Med.* **23**, 416 (2022). 35601074 10.3892/etm.2022.11343 PMC9117961

[151] Elliott, J. et al. Predictive symptoms for COVID-19 in the community: REACT-1 study of over 1 million people. *PLoS Med.* **18**, e1003777 (2021). 34582457 10.1371/journal.pmed.1003777 PMC8478234

[152] Vihta, K. D. et al. Symptoms and severe acute respiratory syndrome coronavirus 2 (SARS-CoV-2) positivity in the general population in the United Kingdom. *Clin. Infect. Dis.* **75**, e329–e337 (2022). 34748629 10.1093/cid/ciab945 PMC8767848

[153] Lippi, G., Nocini, R. & Henry, B. M. Analysis of online search trends suggests that SARS-CoV-2 Omicron (B.1.1.529) variant causes different symptoms. *J. Infect.* **84**, e76–e77 (2022). 35183609 10.1016/j.jinf.2022.02.011 PMC8851877

[154] Arora, S. et al. Literature review of Omicron: a grim reality amidst COVID-19. *Microorganisms* **10**10.3390/microorganisms10020451 (2022). 10.3390/microorganisms10020451 PMC8876621 35208905

[155] Torabi, S. H., Riahi, S. M., Ebrahimzadeh, A. & Salmani, F. Changes in symptoms and characteristics of COVID-19 patients across different variants: two years study using neural network analysis. *BMC Infect. Dis.* **23**, 838 (2023). 38017395 10.1186/s12879-023-08813-9 PMC10683353

[156] Vihta, K. D. et al. Omicron-associated changes in SARS-CoV-2 symptoms in the United Kingdom. *Clin. Infect. Dis.* **76**, e133–e141 (2022). 35917440 10.1093/cid/ciac613 PMC9384604

[157] Yu, W. et al. Proportion of asymptomatic infection and nonsevere disease caused by SARS-CoV-2 Omicron variant: a systematic review and analysis. *J. Med. Virol.* **94**, 5790–5801 (2022). 35961786 10.1002/jmv.28066 PMC9538850

[158] Jeong, Y. J. et al. Current and emerging knowledge in COVID-19. *Radiology* **306**, e222462 (2023). 36625747 10.1148/radiol.222462 PMC9846833

[159] Helms, J. et al. Delirium and encephalopathy in severe COVID-19: a cohort analysis of ICU patients. *Crit. Care* **24**, 491 (2020). 32771053 10.1186/s13054-020-03200-1 PMC7414289

[160] Gupta, A. et al. Extrapulmonary manifestations of COVID-19. *Nat. Med.* **26**, 1017–1032 (2020). 32651579 10.1038/s41591-020-0968-3 PMC11972613

[161] Griffin, D. O. COVID-19: using the right tools at the right time. *Med. Res. Arch.* **10**, 10.18103/mra.v10i8.3041 (2022).

[162] Evert, K. et al. Autopsy findings after long-term treatment of COVID-19 patients with microbiological correlation. *Virchows Arch.* **479**, 97–108 (2021). 33471172 10.1007/s00428-020-03014-0 PMC7816067

[163] Schulte-Schrepping, J. et al. Severe COVID-19 is marked by a dysregulated myeloid cell compartment. *Cell*10.1016/j.cell.2020.08.001 (2020). 10.1016/j.cell.2020.08.001 PMC7405822 32810438

[164] Matschke, J. et al. Neuropathology of patients with COVID-19 in Germany: a post-mortem case series. *Lancet Neurol.* **19**, 919–929 (2020). 33031735 10.1016/S1474-4422(20)30308-2 PMC7535629

[165] Meinhardt, J. et al. Olfactory transmucosal SARS-CoV-2 invasion as a port of central nervous system entry in individuals with COVID-19. *Nat. Neurosci.* **24**, 168–175 (2021). 33257876 10.1038/s41593-020-00758-5

[166] Solomon, T. Neurological infection with SARS-CoV-2—the story so far. *Nat. Rev. Neurol.* **17**, 65–66 (2021). 33414554 10.1038/s41582-020-00453-w PMC7789883

[167] Abu-Raddad, L. J., Chemaitelly, H. & Butt, A. A. & National Study Group for, C.-V. Effectiveness of the BNT162b2 Covid-19 Vaccine against the B.1.1.7 and B.1.351 Variants. *N. Engl. J. Med.* **385**, 187–189 (2021). 33951357 10.1056/NEJMc2104974 PMC8117967

[168] Ackermann, M. et al. Pulmonary vascular endothelialitis, thrombosis, and angiogenesis in Covid-19. *N. Engl. J. Med.* **383**, 120–128 (2020). 32437596 10.1056/NEJMoa2015432 PMC7412750

[169] Teuwen, L. A., Geldhof, V., Pasut, A. & Carmeliet, P. COVID-19: the vasculature unleashed. *Nat. Rev. Immunol.* **20**, 389–391 (2020). 32439870 10.1038/s41577-020-0343-0 PMC7240244

[170] Patone, M. et al. Risks of myocarditis, pericarditis, and cardiac arrhythmias associated with COVID-19 vaccination or SARS-CoV-2 infection. *Nat. Med.* **28**, 410–422 (2022). 34907393 10.1038/s41591-021-01630-0 PMC8863574

[171] Katsoularis, I., Fonseca-Rodriguez, O., Farrington, P., Lindmark, K. & Fors Connolly, A. M. Risk of acute myocardial infarction and ischaemic stroke following COVID-19 in Sweden: a self-controlled case series and matched cohort study. *Lancet* **398**, 599–607 (2021). 34332652 10.1016/S0140-6736(21)00896-5 PMC8321431

[172] Boehmer, T. K. et al. Association between COVID-19 and myocarditis using hospital-based administrative data—United States, March 2020-January 2021. *Morb. Mortal. Wkly Rep.* **70**, 1228–1232 (2021). 10.15585/mmwr.mm7035e5 PMC8422872 34473684

[173] Brogan, M. & Ross, M. J. COVID-19 and kidney disease. *Annu. Rev. Med.* **74**, 1–13 (2023). 36108262 10.1146/annurev-med-042420-104753

[174] Zhang, J. et al. Risk factors for acute kidney injury in COVID-19 patients: an updated systematic review and meta-analysis. *Ren. Fail* **45**, 2170809 (2023). 37021610 10.1080/0886022X.2023.2170809 PMC10081062

[175] Hidayat, A. A. et al. Risk factors and clinical characteristics of acute kidney injury in patients with COVID-19: a systematic review and meta-analysis. *Pathophysiology* **30**, 233–247 (2023). 37218918 10.3390/pathophysiology30020020 PMC10204466

[176] Luo, W., Liu, X., Bao, K. & Huang, C. Ischemic stroke associated with COVID-19: a systematic review and meta-analysis. *J. Neurol.* **269**, 1731–1740 (2022). 34652503 10.1007/s00415-021-10837-7 PMC8517946

[177] Cho, S. M. et al. Neurological manifestations of COVID-19 in adults and children. *Brain* **146**, 1648–1661 (2023). 36087305 10.1093/brain/awac332 PMC9494397

[178] Valyaeva, A. A., Zharikova, A. A. & Sheval, E. V. SARS-CoV-2 cellular tropism and direct multiorgan failure in COVID-19 patients: Bioinformatic predictions, experimental observations, and open questions. *Cell Biol. Int.* **47**, 308–326 (2023). 36229927 10.1002/cbin.11928 PMC9874490

[179] Barbalho, S. M. et al. Organokines in COVID-19: a systematic review. *Cells* **12**10.3390/cells12101349 (2023). 10.3390/cells12101349 PMC10216619 37408184

[180] Yuan, Z., Shao, Z., Ma, L. & Guo, R. Clinical severity of SARS-CoV-2 variants during COVID-19 vaccination: a systematic review and meta-analysis. *Viruses* **15**10.3390/v15101994 (2023). 10.3390/v15101994 PMC10611048 37896770

[181] Abul, Y., Leeder, C. & Gravenstein, S. Epidemiology and clinical presentation of COVID-19 in older adults. *Infect. Dis. Clin. North Am.* **37**, 1–26 (2023). 36805007 10.1016/j.idc.2022.11.001 PMC9633621

[182] Bobrovitz, N. et al. Protective effectiveness of previous SARS-CoV-2 infection and hybrid immunity against the omicron variant and severe disease: a systematic review and meta-regression. * Lancet Infect. Dis.* **23**, 556–567 (2023). ***Systematic review indicating that hybrid immunity (from both infection and vaccination) provides durable protection against severe disease, although protection against reinfection wanes within months***. 36681084 10.1016/S1473-3099(22)00801-5 PMC10014083

[183] Tseng, H. F. et al. Effectiveness of mRNA-1273 against SARS-CoV-2 Omicron and Delta variants. *Nat. Med.* **28**, 1063–1071 (2022). 35189624 10.1038/s41591-022-01753-y PMC9117141

[184] Feikin, D. R. et al. Duration of effectiveness of vaccines against SARS-CoV-2 infection and COVID-19 disease: results of a systematic review and meta-regression. *Lancet* **399**, 924–944 (2022). 35202601 10.1016/S0140-6736(22)00152-0 PMC8863502

[185] Mache, C. et al. SARS-CoV-2 Omicron variant is attenuated for replication in a polarized human lung epithelial cell model. *Commun. Biol.* **5**, 1138 (2022). 36302956 10.1038/s42003-022-04068-3 PMC9610361

[186] Wolter, N. et al. Clinical severity of SARS-CoV-2 Omicron BA.4 and BA.5 lineages compared to BA.1 and Delta in South Africa. *Nat. Commun.* **13**, 5860 (2022). 36195617 10.1038/s41467-022-33614-0 PMC9531215

[187] Wolter, N. et al. Early assessment of the clinical severity of the SARS-CoV-2 omicron variant in South Africa: a data linkage study. *Lancet* **399**, 437–446 (2022). 35065011 10.1016/S0140-6736(22)00017-4 PMC8769664

[188] Bhattacharyya, R. P. & Hanage, W. P. Challenges in inferring intrinsic severity of the SARS-CoV-2 Omicron variant. *N. Engl. J. Med.* **386**, e14 (2022). 35108465 10.1056/NEJMp2119682

[189] Tso, W. W. Y. et al. Severity of SARS-CoV-2 Omicron BA.2 infection in unvaccinated hospitalized children: comparison to influenza and parainfluenza infections. *Emerg. Microbes Infect.* **11**, 1742–1750 (2022). ***Observational study of the severity of Omicron BA.2 in unexposed, unvaccinated, hospitalized children, indicating that in this population Omicron BA.2 was not mild and potentially more neuropathogenic than influenza and parainfluenza viruses***. 35730665 10.1080/22221751.2022.2093135 PMC9258055

[190] Mefsin, Y. M. et al. Epidemiology of infections with SARS-CoV-2 Omicron BA.2 variant, Hong Kong, January-March 2022. *Emerg. Infect. Dis.* **28**, 1856–1858 (2022). 35914518 10.3201/eid2809.220613 PMC9423929

[191] Xie, R. et al. Resurgence of Omicron BA.2 in SARS-CoV-2 infection-naive Hong Kong. *Nat. Commun.* **14**, 2422 (2023). 37105966 10.1038/s41467-023-38201-5 PMC10134727

[192] Brodin, P. SARS-CoV-2 infections in children: understanding diverse outcomes. *Immunity* **55**, 201–209 (2022). 35093190 10.1016/j.immuni.2022.01.014 PMC8769938

[193] Carter, M. J. et al. Peripheral immunophenotypes in children with multisystem inflammatory syndrome associated with SARS-CoV-2 infection. *Nat. Med.* **26**, 1701–1707 (2020). 32812012 10.1038/s41591-020-1054-6

[194] Levy, N. et al. Severity and incidence of multisystem inflammatory syndrome in children during 3 SARS-CoV-2 pandemic waves in Israel. *JAMA* **327**, 2452–2454 (2022). 35588048 10.1001/jama.2022.8025 PMC9121298

[195] Cloete, J. et al. Paediatric hospitalisations due to COVID-19 during the first SARS-CoV-2 omicron (B.1.1.529) variant wave in South Africa: a multicentre observational study. *Lancet Child Adolesc. Health* **6**, 294–302 (2022). 35189083 10.1016/S2352-4642(22)00027-X PMC8856663

[196] Cohen, J. M., Carter, M. J., Cheung, C. R. & Ladhani, S. & Evelina Paediatric Inflammatory Multisystem Syndrome Temporally related to, S.-C.-S. G. Lower risk of multisystem inflammatory syndrome in children with the Delta and Omicron variants of severe acute respiratory syndrome coronavirus 2. *Clin. Infect. Dis.* **76**, e518–e521 (2023). 10.1093/cid/ciac553 PMC9278259 35788276

[197] Sorg, A. L. et al. SARS-CoV-2 variants and the risk of pediatric inflammatory multisystem syndrome temporally associated with SARS-CoV-2 among children in Germany. *Infection* **51**, 729–735 (2023). 36048361 10.1007/s15010-022-01908-6 PMC9435410

[198] Di Gennaro, F. et al. Incidence of long COVID-19 in people with previous SARS-Cov2 infection: a systematic review and meta-analysis of 120,970 patients. *Intern. Emerg. Med.* **18**, 1573–1581 (2023). 10.1007/s11739-022-03164-w PMC9709360 36449260

[199] Lopez-Leon, S. et al. More than 50 long-term effects of COVID-19: a systematic review and meta-analysis. *Sci. Rep.* **11**, 16144 (2021). 34373540 10.1038/s41598-021-95565-8 PMC8352980

[200] Nübel, J. et al. Long COVID – eine Herausforderung für Public Health und Gesundheitsforschung. *Epid. Bull.* **44**, 3–9 (2022).

[201] Davis, H. E., McCorkell, L., Vogel, J. M. & Topol, E. J. Long COVID: major findings, mechanisms and recommendations. *Nat. Rev. Microbiol.* **21**, 133–146 (2023). ***Review on the pathogenesis, clinical features and diagnostics of Long Covid, coauthored by members of the Patient-led Research Collaborative on Long Covid***. 36639608 10.1038/s41579-022-00846-2 PMC9839201

[202] Soriano, J. B. et al. A clinical case definition of post-COVID-19 condition by a Delphi consensus. * Lancet Infect. Dis.* **22**, e102–e107 (2022). 34951953 10.1016/S1473-3099(21)00703-9 PMC8691845

[203] Berlit P. et al. Neurologische Manifestationen bei Covid-19, S1-Leitlinie. https://dgn.org/wp-content/uploads/2020/08/030144_LL_Neurologische_Manifestationen_bei_COVID-19_V3.1.pdf (2021).

[204] Hampshire, A. et al. Cognition and memory after Covid-19 in a large community sample. *N. Engl. J. Med.* **390**, 806–818 (2024). 38416429 10.1056/NEJMoa2311330 PMC7615803

[205] Xu, E., Xie, Y. & Al-Aly, Z. Long-term neurologic outcomes of COVID-19. *Nat. Med.* **28**, 2406–2415 (2022). 36138154 10.1038/s41591-022-02001-z PMC9671811

[206] Monje, M. & Iwasaki, A. The neurobiology of long COVID. *Neuron* **110**, 3484–3496 (2022). 36288726 10.1016/j.neuron.2022.10.006 PMC9537254

[207] Douaud, G. et al. SARS-CoV-2 is associated with changes in brain structure in UK Biobank. *Nature* **604**, 697–707 (2022). 35255491 10.1038/s41586-022-04569-5 PMC9046077

[208] Petersen, M. et al. Brain imaging and neuropsychological assessment of individuals recovered from a mild to moderate SARS-CoV-2 infection. *Proc. Natl. Acad. Sci. USA* **120**, e2217232120 (2023). 37220275 10.1073/pnas.2217232120 PMC10235949

[209] Cecchetti, G. et al. Cognitive, EEG, and MRI features of COVID-19 survivors: a 10-month study. *J. Neurol.* **269**, 3400–3412 (2022). 35249144 10.1007/s00415-022-11047-5 PMC8898558

[210] Fernandez-de-Las-Penas, C. et al. Long-COVID symptoms in individuals infected with different SARS-CoV-2 variants of concern: a systematic review of the literature. *Viruses* **14**10.3390/v14122629 (2022). 10.3390/v14122629 PMC9785120 36560633

[211] Xie, Y., Choi, T. & Al-Aly, Z. Postacute sequelae of SARS-CoV-2 infection in the pre-Delta, Delta, and Omicron eras. *N. Engl. J. Med.* **391**, 515–525 (2024). ***Based on the U.S. Department of Veterans Affairs’ electronic health record system, this study found that while the risk of postacute sequelae of SARS-CoV-2 infection decreased during the omicron era and was reduced with vaccination, it remained notable even among vaccinated individuals infected with Omicron***. 39018527 10.1056/NEJMoa2403211 PMC11687648

[212] O’Sullivan, O. Long-term sequelae following previous coronavirus epidemics. *Clin. Med.* **21**, e68–e70 (2021). 10.7861/clinmed.2020-0204 PMC7850177 33144403

[213] Nalbandian, A. et al. Post-acute COVID-19 syndrome. *Nat. Med.* **27**, 601–615 (2021). 33753937 10.1038/s41591-021-01283-z PMC8893149

[214] Al-Aly, Z., Xie, Y. & Bowe, B. High-dimensional characterization of post-acute sequelae of COVID-19. *Nature* **594**, 259–264 (2021). 33887749 10.1038/s41586-021-03553-9

[215] Al-Aly, Z., Bowe, B. & Xie, Y. Long COVID after breakthrough SARS-CoV-2 infection. *Nat. Med.*10.1038/s41591-022-01840-0 (2022). 10.1038/s41591-022-01840-0 PMC9307472 35614233

[216] Nittas, V. et al. Long COVID through a public health lens: an umbrella review. *Public Health Rev.* **43**, 1604501 (2022). 35359614 10.3389/phrs.2022.1604501 PMC8963488

[217] de Oliveira Almeida, K. et al. A systematic review on physical function, activities of daily living and health-related quality of life in COVID-19 survivors. *Chronic Illn.* **19**, 279–303 (2023). 10.1177/17423953221089309 PMC9006095 35404175

[218] Xie, Y., Xu, E., Bowe, B. & Al-Aly, Z. Long-term cardiovascular outcomes of COVID-19. *Nat. Med.* **28**, 583–590 (2022). 35132265 10.1038/s41591-022-01689-3 PMC8938267

[219] Xie, Y. & Al-Aly, Z. Risks and burdens of incident diabetes in long COVID: a cohort study. *Lancet Diab. Endocrinol.* **10**, 311–321 (2022). 10.1016/S2213-8587(22)00044-4 PMC8937253 35325624

[220] Taylor, K. et al. Incidence of diabetes after SARS-CoV-2 infection in England and the implications of COVID-19 vaccination: a retrospective cohort study of 16 million people. *Lancet Diab. Endocrinol.* **12**, 558–568 (2024). 10.1016/S2213-8587(24)00159-1 PMC7617111 39054034

[221] Yende, S. & Parikh, C. R. Long COVID and kidney disease. *Nat. Rev. Nephrol.* **17**, 792–793 (2021). 34504319 10.1038/s41581-021-00487-3 PMC8427150

[222] Taquet, M., Geddes, J. R., Husain, M., Luciano, S. & Harrison, P. J. 6-month neurological and psychiatric outcomes in 236 379 survivors of COVID-19: a retrospective cohort study using electronic health records. *Lancet Psychiatry* **8**, 416–427 (2021). 33836148 10.1016/S2215-0366(21)00084-5 PMC8023694

[223] Xie, Y., Xu, E. & Al-Aly, Z. Risks of mental health outcomes in people with covid-19: cohort study. *BMJ* **376**, e068993 (2022). 35172971 10.1136/bmj-2021-068993 PMC8847881

[224] Fajnzylber, J. et al. SARS-CoV-2 viral load is associated with increased disease severity and mortality. *Nat. Commun.* **11**, 5493 (2020). 33127906 10.1038/s41467-020-19057-5 PMC7603483

[225] Pujadas, E. et al. SARS-CoV-2 viral load predicts COVID-19 mortality. *Lancet Respir. Med.* **8**, e70 (2020). 32771081 10.1016/S2213-2600(20)30354-4 PMC7836878

[226] Pang, X. et al. Emerging severe acute respiratory syndrome coronavirus 2 mutation hotspots associated with clinical outcomes and transmission. *Front. Microbiol.* **12**, 753823 (2021). 34733263 10.3389/fmicb.2021.753823 PMC8558435

[227] Dao, T. L. et al. SARS-CoV-2 infectivity and severity of COVID-19 according to SARS-CoV-2 variants: current evidence. *J. Clin. Med.* **10**10.3390/jcm10122635 (2021). 10.3390/jcm10122635 PMC8232800 34203844

[228] Wagner, C. et al. Positive selection underlies repeated knockout of ORF8 in SARS-CoV-2 evolution. *Nat. Commun.* **15**, 3207 (2024). 38615031 10.1038/s41467-024-47599-5 PMC11016114

[229] Ichikawa, T. et al. Mutations in the nonstructural proteins of SARS-CoV-2 may contribute to adverse clinical outcome in patients with COVID-19. *Int. J. Infect. Dis.* **122**, 123–129 (2022). 35562044 10.1016/j.ijid.2022.05.010 PMC9088090

[230] Scripps Institute. *B.1.617.2 Lineage Report on* https://outbreak.info/situation-reports?pango=B.1.617.2&selected=IND_IN-MH (2021).

[231] Chen, D. Y. et al. Spike and nsp6 are key determinants of SARS-CoV-2 Omicron BA.1 attenuation. *Nature* **615**, 143–150 (2023). 36630998 10.1038/s41586-023-05697-2

[232] Martins, M. et al. The SARS-CoV-2 Spike is a virulence determinant and plays a major role on the attenuated phenotype of Omicron virus in a feline model of infection. *J. Virol.* **98**, e0190223 (2024). 38421180 10.1128/jvi.01902-23 PMC10949471

[233] Sangare, K. et al. Combined mutations in nonstructural protein 14, envelope, and membrane proteins mitigate the neuropathogenicity of SARS-CoV-2 Omicron BA.1 in K18-hACE2 mice. *mSphere* **10**, e0072624 (2025). 39660912 10.1128/msphere.00726-24 PMC11774043

[234] McGrath, M. E. et al. SARS-CoV-2 ORF8 modulates lung inflammation and clinical disease progression. *PLoS Pathog.* **20**, e1011669 (2024). 38781259 10.1371/journal.ppat.1011669 PMC11152254

[235] Lok, L. S. C. et al. Long COVID across SARS-CoV-2 variants: Clinical features, pathogenesis, and future directions. *MedComm Future Med.* **3**, e70004 (2024).

[236] Diexer, S. et al. Association between virus variants, vaccination, previous infections, and post-COVID-19 risk. *Int. J. Infect. Dis.* **136**, 14–21 (2023). 37634619 10.1016/j.ijid.2023.08.019

[237] Du, M., Ma, Y., Deng, J., Liu, M. & Liu, J. Comparison of long COVID-19 caused by different SARS-CoV-2 strains: a systematic review and meta-analysis. *Int. J. Environ. Res. Public Health* **19**10.3390/ijerph192316010 (2022). 10.3390/ijerph192316010 PMC9736973 36498103

[238] Hernandez-Aceituno, A., Garcia-Hernandez, A. & Larumbe-Zabala, E. COVID-19 long-term sequelae: Omicron versus Alpha and Delta variants. *Infect. Dis. Now.* **53**, 104688 (2023). 36858287 10.1016/j.idnow.2023.104688 PMC9970656

[239] Percze, A. R. et al. Fatigue, sleepiness and sleep quality are SARS-CoV-2 variant independent in patients with long COVID symptoms. *Inflammopharmacology* **31**, 2819–2825 (2023). 37020055 10.1007/s10787-023-01190-4 PMC10075170

[240] Saigal, A. et al. Cross-sectional study evaluating the impact of SARS-CoV-2 variants on Long COVID outcomes in UK hospital survivors. *BMJ Open Respir. Res.* **10**10.1136/bmjresp-2023-001667 (2023). 10.1136/bmjresp-2023-001667 PMC10401240 37536948

[241] Cao, Y. et al. Omicron escapes the majority of existing SARS-CoV-2 neutralizing antibodies. *Nature* **602**, 657–663 (2022). 35016194 10.1038/s41586-021-04385-3 PMC8866119

[242] VanBlargan, L. A. et al. An infectious SARS-CoV-2 B.1.1.529 Omicron virus escapes neutralization by therapeutic monoclonal antibodies. *Nat. Med*. 10.1038/s41591-021-01678-y (2022). 10.1038/s41591-021-01678-y PMC8767531 35046573

[243] Zhou, H., Dcosta, B. M., Landau, N. R. & Tada, T. Resistance of SARS-CoV-2 Omicron BA.1 and BA.2 variants to vaccine-elicited sera and therapeutic monoclonal antibodies. *Viruses* **14**10.3390/v14061334 (2022). 10.3390/v14061334 PMC9228817 35746806

[244] Iketani, S. et al. Antibody evasion properties of SARS-CoV-2 Omicron sublineages. *Nature* **604**, 553–556 (2022). 35240676 10.1038/s41586-022-04594-4 PMC9021018

[245] Xiaoliang, X. et al. BA.2.12.1, BA.4 and BA.5 escape antibodies elicited by Omicron BA.1. 10.21203/rs.3.rs-1611421/v1 (2022).

[246] European Medicines Agency. ETF statement on the loss of activity of anti-spike protein monoclonal antibodies due to emerging SARS-CoV-2 variants. https://www.ema.europa.eu/en/documents/other/etf-statement-loss-activity-anti-spike-protein-monoclonal-antibodies-due-emerging-sars-cov-2-variants-december-2024-update_en.pdf (2024).

[247] Stanford HIVDB Team. Stanford Coronavirus Antiviral & Resistance Database. https://covdb.stanford.edu/ (2020–2024).

[248] Tzou, P. L., Tao, K., Pond, S. L. K. & Shafer, R. W. Coronavirus Resistance Database (CoV-RDB): SARS-CoV-2 susceptibility to monoclonal antibodies, convalescent plasma, and plasma from vaccinated persons. *PLoS ONE* **17**, e0261045 (2022). 35263335 10.1371/journal.pone.0261045 PMC8906623

[249] Costacurta, F. et al. A comprehensive study of SARS-CoV-2 main protease (M(pro)) inhibitor-resistant mutants selected in a VSV-based system. *bioRxiv*10.1101/2023.09.22.558628 (2023). 10.1371/journal.ppat.1012522 PMC11407635 39259728

[250] Cele, S. et al. SARS-CoV-2 prolonged infection during advanced HIV disease evolves extensive immune escape. *Cell Host Microbe* **30**, 154–162.e155 (2022). ***SARS-CoV-2 evolution in an immunocompromised individual with advanced HIV in South Africa led to the emergence of mutations resembling those in Omicron and other variants, demonstrating significant immune escape from vaccines and prior Delta infection, highlighting the potential role of such hosts in the development of vaccine-resistant strains***. 35120605 10.1016/j.chom.2022.01.005 PMC8758318

[251] Aydillo, T. et al. Shedding of viable SARS-CoV-2 after immunosuppressive therapy for cancer. *N. Engl. J. Med.* **383**, 2586–2588 (2020). 33259154 10.1056/NEJMc2031670 PMC7722690

[252] Huang, F. et al. Identifying COVID-19 severity-related SARS-CoV-2 mutation using a machine learning method. *Life* **12**10.3390/life12060806 (2022). 10.3390/life12060806 PMC9225528 35743837

[253] Alanagreh, L., Alzoughool, F. & Atoum, M. The Human Coronavirus Disease COVID-19: its origin, characteristics, and insights into potential drugs and its mechanisms. *Pathogens* **9**10.3390/pathogens9050331 (2020). 10.3390/pathogens9050331 PMC7280997 32365466

[254] Zhang, Q. et al. Molecular mechanism of interaction between SARS-CoV-2 and host cells and interventional therapy. *Signal Transduct. Target Ther.* **6**, 233 (2021). 34117216 10.1038/s41392-021-00653-w PMC8193598

[255] Gitman, M. R., Shaban, M. V., Paniz-Mondolfi, A. E. & Sordillo, E. M. Laboratory diagnosis of SARS-CoV-2 pneumonia. *Diagnostics* **11**10.3390/diagnostics11071270 (2021). 10.3390/diagnostics11071270 PMC8306256 34359353

[256] Rajpal, V. R. et al. A comprehensive account of SARS-CoV-2 genome structure, incurred mutations, lineages and COVID-19 vaccination program. *Future Virol.*10.2217/fvl-2021-0277 (2022). 10.2217/fvl-2021-0277 PMC9203033 35747328

[257] Wu, F. et al. A new coronavirus associated with human respiratory disease in China. *Nature* **579**, 265–269 (2020). 32015508 10.1038/s41586-020-2008-3 PMC7094943

[258] Yurkovetskiy, L. et al. Structural and functional analysis of the D614G SARS-CoV-2 spike protein variant. *Cell* **183**, 739–751.e738 (2020). 32991842 10.1016/j.cell.2020.09.032 PMC7492024

[259] Zhang, L. et al. SARS-CoV-2 spike-protein D614G mutation increases virion spike density and infectivity. *Nat. Commun.* **11**, 6013 (2020). 33243994 10.1038/s41467-020-19808-4 PMC7693302

[260] Hou, Y. J. et al. SARS-CoV-2 D614G variant exhibits efficient replication ex vivo and transmission in vivo. *Science* **370**, 1464–1468 (2020). 33184236 10.1126/science.abe8499 PMC7775736

[261] Plante, J. A. et al. Spike mutation D614G alters SARS-CoV-2 fitness. *Nature*10.1038/s41586-020-2895-3 (2020). 10.1038/s41586-020-2895-3 PMC8158177 33106671

[262] Volz, E. et al. Evaluating the effects of SARS-CoV-2 spike mutation D614G on transmissibility and pathogenicity. *Cell* **184**, 64–75.e11 (2021). 33275900 10.1016/j.cell.2020.11.020 PMC7674007

[263] Rambaut, A. et al. Preliminary genomic characterisation of an emergent SARS-CoV-2 lineage in the UK defined by a novel set of spike mutations. https://virological.org/t/preliminary-genomic-characterisation-of-an-emergent-sars-cov-2-lineage-in-the-uk-defined-by-a-novel-set-of-spike-mutations/563 (2020).

[264] Public Health England. Investigation of novel SARS-COV-2 variant: Variant of Concern 202012/01. PHE Technical Briefing (2020).

[265] New and Emerging Respiratory Virus Threats Advisory Group. NERVTAG note on B.1.1.7 severity. (2021).

[266] Vöhringer, H. et al. Lineage-specific growth of SARS-CoV-2 B.1.1.7 during the English national lockdown. https://virological.org/t/lineage-specific-growth-of-sars-cov-2-b-1-1-7-during-the-english-national-lockdown/575 (2020).

[267] Public Health England. Investigation of novel SARS-CoV-2 variant. Variant of Concern 202012/01. Technical briefing 3. https://assets.publishing.service.gov.uk/government/uploads/system/uploads/attachment_data/file/950823/Variant_of_Concern_VOC_202012_01_Technical_Briefing_3_-_England.pdf (2021).

[268] Jones, T. C. et al. Estimating infectiousness throughout SARS-CoV-2 infection course. *Science* **373**10.1126/science.abi5273 (2021). 10.1126/science.abi5273 PMC9267347 34035154

[269] Kidd, M. et al. S-variant SARS-CoV-2 lineage B1.1.7 is associated with significantly higher viral loads in samples tested by ThermoFisher TaqPath RT-qPCR. *J. Infect. Dis*. 10.1093/infdis/jiab082 (2021). 10.1093/infdis/jiab082 PMC7928763 33580259

[270] Kissler, S. M. et al. Viral dynamics of SARS-CoV-2 variants in vaccinated and unvaccinated persons. *N. Engl. J. Med.* **385**, 2489–2491 (2021). ***Longitudinal study of SARS-CoV-2 viral dynamics in individuals (within the “NBA bubble”) infected with different variants and vaccination statuses, which found no significant differences in peak viral load or infection duration between the Alpha and Delta variants or between vaccinated and unvaccinated individuals, though vaccinated individuals cleared the virus faster, highlighting the need for more diverse and comprehensive studies to better understand these dynamics***. 34941024 10.1056/NEJMc2102507 PMC8693673

[271] Walker, A. S. et al. Increased infections, but not viral burden, with a new SARS-CoV-2 variant. *medRxiv*10.1101/2021.01.13.21249721 (2021).

[272] Niemeyer, D. et al. SARS-CoV-2 variant Alpha has a spike-dependent replication advantage over the ancestral B.1 strain in human cells with low ACE2 expression. *PLoS Biol.* **20**, e3001871 (2022). 36383605 10.1371/journal.pbio.3001871 PMC9710838

[273] Borges, V. et al. Tracking SARS-CoV-2 VOC 202012/01 (lineage B.1.1.7) dissemination in Portugal: insights from nationwide RT-PCR Spike gene drop out data. *Virological.org* https://virological.org/t/tracking-sars-cov-2-voc-202012-01-lineage-b-1-1-7-dissemination-in-portugal-insights-from-nationwide-rt-pcr-spike-gene-drop-out-data/600 (2021).

[274] Statens Serum Institut. Nyt kontakttal for virusvariant B.1.1.7. https://www.ssi.dk/aktuelt/nyheder/2021/nyt-kontakttal-for-virusvariant-b117 (2021).

[275] Public Health England. SARS-CoV-2 variants of concern and variants under investigation in England. Technical briefing 10. https://assets.publishing.service.gov.uk/government/uploads/system/uploads/attachment_data/file/984274/Variants_of_Concern_VOC_Technical_Briefing_10_England.pdf (2021).

[276] Public Health England. SARS-CoV-2 variants of concern and variants under investigationin England. Technical briefing 14. https://assets.publishing.service.gov.uk/government/uploads/system/uploads/attachment_data/file/991343/Variants_of_Concern_VOC_Technical_Briefing_14.pdf (2021).

[277] Fisman, D. N. & Tuite, A. R. Evaluation of the relative virulence of novel SARS-CoV-2 variants: a retrospective cohort study in Ontario, Canada. *CMAJ* **193**, E1619–E1625 (2021). 34610919 10.1503/cmaj.211248 PMC8562985

[278] Sheikh, A. et al. SARS-CoV-2 Delta VOC in Scotland: demographics, risk of hospital admission, and vaccine effectiveness. *Lancet* **397**, 2461–2462 (2021). 34139198 10.1016/S0140-6736(21)01358-1 PMC8201647

[279] Motozono, C. et al. SARS-CoV-2 spike L452R variant evades cellular immunity and increases infectivity. *Cell Host Microbe* **29**, 1124–1136.e1111 (2021). 34171266 10.1016/j.chom.2021.06.006 PMC8205251

[280] Peacock, T. P. et al. The SARS-CoV-2 variants associated with infections in India, B.1.617, show enhanced spike cleavage by furin. *bioRxiv*10.1101/2021.05.28.446163 (2021).

[281] Mlcochova, P. et al. SARS-CoV-2 B.1.617.2 Delta variant replication and immune evasion. *Nature* **599**, 114–119 (2021). 34488225 10.1038/s41586-021-03944-y PMC8566220

[282] Saito, A. et al. Enhanced fusogenicity and pathogenicity of SARS-CoV-2 Delta P681R mutation. *Nature* **602**, 300–306 (2022). 34823256 10.1038/s41586-021-04266-9 PMC8828475

[283] Twohig, K. A. et al. Hospital admission and emergency care attendance risk for SARS-CoV-2 delta (B.1.617.2) compared with alpha (B.1.1.7) variants of concern: a cohort study. * Lancet Infect. Dis.* **22**, 35–42 (2022). 34461056 10.1016/S1473-3099(21)00475-8 PMC8397301

[284] Ong, S. W. X. et al. Clinical and virological features of severe acute respiratory syndrome coronavirus 2 (SARS-CoV-2) variants of concern: a retrospective cohort study comparing B.1.1.7 (Alpha), B.1.351 (Beta), and B.1.617.2 (Delta). *Clin. Infect. Dis.* **75**, e1128–e1136 (2022). 34423834 10.1093/cid/ciab721 PMC8522361

[285] Fisman, D. N. & Tuite, A. R. Age-specific changes in virulence associated with severe acute respiratory syndrome coronavirus 2 (SARS-CoV-2) variants of concern. *Clin. Infect. Dis.* **75**, e69–e75 (2022). 35234859 10.1093/cid/ciac174 PMC9047153

[286] Liu, C. et al. Reduced neutralization of SARS-CoV-2 B.1.617 by vaccine and convalescent serum. *Cell*10.1016/j.cell.2021.06.020 (2021). 10.1016/j.cell.2021.06.020 PMC8218332 34242578

[287] Planas, D. et al. Reduced sensitivity of SARS-CoV-2 variant Delta to antibody neutralization. *Nature*10.1038/s41586-021-03777-9 (2021). 10.1038/s41586-021-03777-9 34237773

[288] Nasreen, S. et al. Effectiveness of COVID-19 vaccines against symptomatic SARS-CoV-2 infection and severe outcomes with variants of concern in Ontario. *Nat. Microbiol*. 10.1038/s41564-021-01053-0 (2022). 10.1038/s41564-021-01053-0 35132198

[289] Lopez Bernal, J. et al. Effectiveness of Covid-19 vaccines against the B.1.617.2 (Delta) variant. *N. Engl. J. Med*. 10.1056/NEJMoa2108891 (2021). 10.1056/NEJMoa2108891 PMC8314739 34289274

[290] Stowe, J. et al. Effectiveness of COVID-19 vaccines against hospital admission with the Delta (B.1.617.2) variant. PHE Public Library. https://khub.net/web/phe-national/public-library/-/document_library/v2WsRK3ZlEig/view_file/479607329?_com_liferay_document_library_web_portlet_DLPortlet_INSTANCE_v2WsRK3ZlEig_redirect=https%3A%2F%2Fkhub.net%3A443%2Fweb%2Fphe-national%2Fpublic-library%2F-%2Fdocument_library%2Fv2WsRK3ZlEig%2Fview%2F479607266 (2021).

[291] Wang, P. et al. Antibody Resistance of SARS-CoV-2 variants B.1.351 and B.1.1.7. *Nature*10.1038/s41586-021-03398-2 (2021). 10.1038/s41586-021-03398-2 33684923

[292] Greaney, A. J. et al. Comprehensive mapping of mutations in the SARS-CoV-2 receptor-binding domain that affect recognition by polyclonal human plasma antibodies. *Cell Host Microbe* **29**, 463–476.e466 (2021). 33592168 10.1016/j.chom.2021.02.003 PMC7869748

[293] Weisblum, Y. et al. Escape from neutralizing antibodies by SARS-CoV-2 spike protein variants. *Elife* **9**10.7554/eLife.61312 (2020). 10.7554/eLife.61312 PMC7723407 33112236

[294] Andreano, E. et al. SARS-CoV-2 escape from a highly neutralizing COVID-19 convalescent plasma. *Proc. Natl. Acad. Sci. USA* **118**10.1073/pnas.2103154118 (2021). 10.1073/pnas.2103154118 PMC8433494 34417349

[295] Cele, S. et al. Escape of SARS-CoV-2 501Y.V2 from neutralization by convalescent plasma. *Nature* **593**, 142–146 (2021). 33780970 10.1038/s41586-021-03471-w PMC9867906

[296] Wibmer, C. K. et al. SARS-CoV-2 501Y.V2 escapes neutralization by South African COVID-19 donor plasma. *Nat. Med.*10.1038/s41591-021-01285-x (2021). 10.1038/s41591-021-01285-x 33654292

[297] Shinde, V. et al. Efficacy of NVX-CoV2373 Covid-19 vaccine against the B.1.351 Variant. *N. Engl. J. Med.* **384**, 1899–1909 (2021). ***The ChAdOx1 nCoV-19 (AZD1222) vaccine was found to be safe but showed limited efficacy (10.4%) against mild-to-moderate COVID-19 caused by the B.1.351 variant in a South African trial, highlighting the need for vaccine adaptations to address emerging SARS-CoV-2 variants.*** 10.1056/NEJMoa2103055 PMC8091623 33951374

[298] Madhi, S. A. et al. Efficacy of the ChAdOx1 nCoV-19 Covid-19 vaccine against the B.1.351 variant. *N. Engl. J. Med*. 10.1056/NEJMoa2102214 (2021). 10.1056/NEJMoa2102214 PMC7993410 33725432

[299] Choi, A. et al. Safety and immunogenicity of SARS-CoV-2 variant mRNA vaccine boosters in healthy adults: an interim analysis. *Nat. Med.* **27**, 2025–2031 (2021). 34526698 10.1038/s41591-021-01527-y PMC8604720

[300] Pearson, C. A. B. et al. Estimates of severity and transmissibility of novel SARS-CoV-2 variant 501Y.V2 in South Africa. *CMMID Repository* https://cmmid.github.io/topics/covid19/sa-novel-variant.html (2021).

[301] Taylor, L. Covid-19: Is Manaus the final nail in the coffin for natural herd immunity?. *BMJ* **372**, n394 (2021). 33579721 10.1136/bmj.n394

[302] Faria, N. R. et al. Genomic characterisation of an emergent SARS-CoV-2 lineage in Manaus: preliminary findings. https://virological.org/t/genomic-characterisation-of-an-emergent-sars-cov-2-lineage-in-manaus-preliminary-findings/586 (2021).

[303] Buss, L. F. et al. Three-quarters attack rate of SARS-CoV-2 in the Brazilian Amazon during a largely unmitigated epidemic. *Science* **371**, 288–292 (2021). 33293339 10.1126/science.abe9728 PMC7857406

[304] Wang, P. et al. Increased resistance of SARS-CoV-2 variant P.1 to antibody neutralization. *Cell Host Microbe* **29**, 747–751.e744 (2021). 33887205 10.1016/j.chom.2021.04.007 PMC8053237

[305] Wolter, N., Jassat, W., group, D.-G. A., von Gottberg, A. & Cohen, C. Clinical severity of Omicron sub-lineage BA.2 compared to BA.1 in South Africa. *medRxiv*10.1101/2022.02.17.22271030 (2022). 10.1016/S0140-6736(22)00981-3 PMC9246473 35780802

[306] Lyngse, F. P. et al. Household transmission of SARS-CoV-2 Omicron variant of concern subvariants BA.1 and BA.2 in Denmark. *Nat. Commun.* **13**, 5760 (2022). 36180438 10.1038/s41467-022-33498-0 PMC9524324

[307] Buda, S. et al. Wochenberichte der AGI. https://influenza.rki.de/Wochenberichte.aspx (2022).

[308] World Health Organization. TAG-VE statement on Omicron sublineages BQ.1 and XBB. https://www.who.int/news/item/27-10-2022-tag-ve-statement-on-omicron-sublineages-bq.1-and-xbb (2022).

[309] Davis-Gardner, M. E. et al. mRNA bivalent booster enhances neutralization against BA.2.75.2 and BQ.1.1. *bioRxiv*10.1101/2022.10.31.514636 (2022).

[310] O’Toole, A. et al. Assignment of epidemiological lineages in an emerging pandemic using the pangolin tool. *Virus Evol.* **7**, veab064 (2021). 34527285 10.1093/ve/veab064 PMC8344591

[311] Wilks, S. H. et al. Mapping SARS-CoV-2 antigenic relationships and serological responses. *Science* **382**, eadj0070 (2023). ***This study demonstrates that a small number of key substitutions in the SARS-CoV-2 spike protein drive significant antigenic variation among variants, affecting immune escape and altering immune response targets based on prior infections or vaccinations; understanding these dynamics is critical for developing vaccines that maintain high efficacy across diverse populations with varying immunity profiles***. 37797027 10.1126/science.adj0070 PMC12145880

[312] van der Straten, K. et al. Antigenic cartography using sera from sequence-confirmed SARS-CoV-2 variants of concern infections reveals antigenic divergence of Omicron. *Immunity* **55**, 1725–1731.e1724 (2022). 35973428 10.1016/j.immuni.2022.07.018 PMC9353602

[313] Mykytyn, A. Z. et al. Antigenic cartography of SARS-CoV-2 reveals that Omicron BA.1 and BA.2 are antigenically distinct. *Sci. Immunol.* **7**, eabq4450 (2022). 35737747 10.1126/sciimmunol.abq4450 PMC9273038

[314] UK Health Security Agency. SARS-CoV-2 variants of concern and variants under investigation in England: Technical Briefing 31. (2021).

[315] Espenhain, L. et al. Epidemiological characterisation of the first 785 SARS-CoV-2 Omicron variant cases in Denmark, December 2021. *Euro Surveill.* **26**10.2807/1560-7917.ES.2021.26.50.2101146 (2021). 10.2807/1560-7917.ES.2021.26.50.2101146 PMC8728489 34915977

[316] Brandal, L. T. et al. Outbreak caused by the SARS-CoV-2 Omicron variant in Norway, November to December 2021. *Euro Surveill.* **26**10.2807/1560-7917.ES.2021.26.50.2101147 (2021). 10.2807/1560-7917.ES.2021.26.50.2101147 PMC8728491 34915975

[317] Eggink, D. et al. Increased risk of infection with SARS-CoV-2 Omicron BA.1 compared with Delta in vaccinated and previously infected individuals, the Netherlands, 22 November 2021 to 19 January 2022. *Euro Surveill.* **27**10.2807/1560-7917.ES.2022.27.4.2101196 (2022). 10.2807/1560-7917.ES.2022.27.4.2101196 PMC8796294 35086609

[318] Andrews, N. et al. Covid-19 vaccine effectiveness against the Omicron (B.1.1.529) variant. *N. Engl. J. Med.* **386**, 1532–1546 (2022). ***This study found that two doses of the ChAdOx1 nCoV-19 or BNT162b2 vaccines provided limited protection against symptomatic disease caused by the Omicron variant, while booster doses of BNT162b2 or mRNA-1273 significantly increased vaccine effectiveness, though this protection waned over time.*** 10.1056/NEJMoa2119451 PMC8908811 35249272

[319] UK Health Security Agency. Technical briefing: update on hospitalisation and vaccine effectiveness for Omicron VOC-21NOV-01 (B.1.1.529). https://assets.publishing.service.gov.uk/government/uploads/system/uploads/attachment_data/file/1044481/Technical-Briefing-31-Dec-2021-Omicron_severity_update.pdf (2021).

[320] Chemaitelly, H. et al. Duration of protection of BNT162b2 and mRNA-1273 COVID-19 vaccines against symptomatic SARS-CoV-2 Omicron infection in Qatar. *medRxiv*10.1101/2022.02.07.22270568 (2022).

[321] Tseng, H. F. et al. Effectiveness of mRNA-1273 against SARS-CoV-2 Omicron and Delta variants. *Nat. Med*. 10.1038/s41591-022-01753-y (2022). 10.1038/s41591-022-01753-y PMC9117141 35189624

[322] Wang, Q. et al. Deep immunological imprinting due to the ancestral spike in the current bivalent COVID-19 vaccine. *Cell Rep. Med.* **4**, 101258 (2023). 37909042 10.1016/j.xcrm.2023.101258 PMC10694617

[323] Monto, A. S., Malosh, R. E., Petrie, J. G. & Martin, E. T. The doctrine of original antigenic sin: separating good from evil. *J. Infect. Dis.* **215**, 1782–1788 (2017). 28398521 10.1093/infdis/jix173 PMC5853211

[324] Wang, Q. et al. SARS-CoV-2 neutralising antibodies after a second BA.5 bivalent booster. *Lancet* **402**, 1827–1828 (2023). 37922920 10.1016/S0140-6736(23)02278-X PMC13141638

[325] World Health Organization. Statement on the antigen composition of COVID-19 vaccines. https://www.who.int/news/item/18-05-2023-statement-on-the-antigen-composition-of-covid-19-vaccines (2023).

[326] Yisimayi, A. et al. Repeated Omicron exposures override ancestral SARS-CoV-2 immune imprinting. *Nature* **625**, 148–156 (2024). 37993710 10.1038/s41586-023-06753-7 PMC10764275

[327] UK Health Security Agency. SARS-CoV-2 variants of concern and variants under investigation in England. Technical Briefing 34. (2022).

[328] Society of Nuclear Medicine and Molecular Imaging (SNMMI). SNMMI statement: possible effect of Omicron infection on FDG PET/CT scans. (2022).

[329] Iuliano, A. D. et al. Trends in disease severity and health care utilization during the early Omicron variant period compared with previous SARS-CoV-2 high transmission periods—United States, December 2020-January 2022. *Morb. Mortal. Wkly Rep.* **71**, 146–152 (2022). 10.15585/mmwr.mm7104e4 PMC9351529 35085225

[330] Nyberg, T. et al. Comparative analysis of the risks of hospitalisation and death associated with SARS-CoV-2 omicron (B.1.1.529) and delta (B.1.617.2) variants in England: a cohort study. * Lancet* **399**, 1303–1312 (2022). 35305296 10.1016/S0140-6736(22)00462-7 PMC8926413

[331] Wang, L. et al. Comparison of outcomes from COVID infection in pediatric and adult patients before and after the emergence of Omicron. *medRxiv*10.1101/2021.12.30.21268495 (2022).

[332] Lin, B. et al. Clinical and radiological characteristics of pediatric COVID-19 before and after the Omicron outbreak: a multi-center study. *Front. Pediatr.* **11**, 1172111 (2023). 37664548 10.3389/fped.2023.1172111 PMC10470622

[333] Brady, D. K. et al. A guide to COVID-19 antiviral therapeutics: a summary and perspective of the antiviral weapons against SARS-CoV-2 infection. *FEBS J*. 10.1111/febs.16662 (2022). 10.1111/febs.16662 PMC9874604 36266238

[334] Shimizu, R. et al. Safety, tolerability, and pharmacokinetics of the novel antiviral agent ensitrelvir fumaric acid, a SARS-CoV-2 3CL protease inhibitor, in healthy adults. *Antimicrob. Agents Chemother.* **66**, e0063222 (2022). 36094202 10.1128/aac.00632-22 PMC9578392

[335] Mukae, H. et al. A randomized phase 2/3 study of ensitrelvir, a novel oral SARS-CoV-2 3C-like protease inhibitor, in Japanese patients with mild-to-moderate COVID-19 or asymptomatic SARS-CoV-2 infection: results of the phase 2a part. *Antimicrob. Agents Chemother.* **66**, e0069722 (2022). 36098519 10.1128/aac.00697-22 PMC9578433

[336] Antar, A. A. R. & Peluso, M. J. CROI 2023: acute and post-acute COVID-19. *Top. Antivir. Med.* **31**, 493–509 (2023). 37315513 PMC10266867

[337] Xie, Y., Choi, T. & Al-Aly, Z. Association of treatment with nirmatrelvir and the risk of post-COVID-19 condition. *JAMA Intern. Med.* **183**, 554–564 (2023). 36951829 10.1001/jamainternmed.2023.0743 PMC10037200

[338] Al-Aly, Z. SARS-CoV-2 antivirals and post-COVID-19 condition. * Lancet Infect. Dis.* **25**, 6–8 (2025). 39265592 10.1016/S1473-3099(24)00436-5

[339] Beigel, J. H. et al. Remdesivir for the treatment of Covid-19—final report. *N. Engl. J. Med.* **383**, 1813–1826 (2020). ***This study demonstrated that remdesivir significantly shortened the median time to recovery from 15 days to 10 days in adults hospitalized with COVID-19 and lower respiratory tract infections compared to placebo, with a 29% faster recovery rate, and showed a trend towards reduced mortality by day 29, suggesting remdesivir’s efficacy as an antiviral treatment for COVID-19.*** 10.1056/NEJMoa2007764 PMC7262788 32445440

[340] Gottlieb, R. L. et al. Early remdesivir to prevent progression to severe Covid-19 in outpatients. *N. Engl. J. Med.* **386**, 305–315 (2022). 34937145 10.1056/NEJMoa2116846 PMC8757570

[341] Meyerowitz, E. A. & Li, Y. Review: The landscape of antiviral therapy for COVID-19 in the era of widespread population immunity and Omicron-lineage viruses. *Clin. Infect. Dis.* **78**, 908–917 (2024). 37949817 10.1093/cid/ciad685 PMC11487108

[342] Tian, L. et al. Molnupiravir and its antiviral activity against COVID-19. *Front. Immunol.* **13**, 855496 (2022). 35444647 10.3389/fimmu.2022.855496 PMC9013824

[343] Santani, B. G., LeBlanc, B. W. & Thakare, R. P. Molnupiravir for the treatment of COVID-19. *Drugs Today* **58**, 335–350 (2022). 10.1358/dot.2022.58.7.3419558 35851869

[344] Jayk Bernal, A. et al. Molnupiravir for oral treatment of Covid-19 in nonhospitalized patients. *N. Engl. J. Med.* **386**, 509–520 (2022). 34914868 10.1056/NEJMoa2116044 PMC8693688

[345] Schilling, W. H. K. et al. Antiviral efficacy of molnupiravir versus ritonavir-boosted nirmatrelvir in patients with early symptomatic COVID-19 (PLATCOV): an open-label, phase 2, randomised, controlled, adaptive trial. * Lancet Infect. Dis.* **24**, 36–45 (2024). 37778363 10.1016/S1473-3099(23)00493-0 PMC7615401

[346] Butler, C. C. et al. Molnupiravir plus usual care versus usual care alone as early treatment for adults with COVID-19 at increased risk of adverse outcomes (PANORAMIC): an open-label, platform-adaptive randomised controlled trial. *Lancet* **401**, 281–293 (2023). 36566761 10.1016/S0140-6736(22)02597-1 PMC9779781

[347] Sanderson, T. et al. A molnupiravir-associated mutational signature in global SARS-CoV-2 genomes. *Nature* **623**, 594–600 (2023). ***This study identified a distinct mutational signature in SARS-CoV-2 sequences that was strongly associated with molnupiravir treatment, raising concerns about the potential onward transmission of molnupiravir-mutated viruses, especially in cases where infections are not fully cleared***. 37748513 10.1038/s41586-023-06649-6 PMC10651478

[348] European Medicines Agency. Withdrawal of application for the marketing authorisation of Lagevrio (molnupiravir). https://www.ema.europa.eu/en/medicines/human/EPAR/lagevrio#ema-inpage-item-key-facts (2023).

