# Supplementary material for: A public health perspective of SARS-CoV-2 evolution and surveillance strategies in Germany from 2020 to 2023
Source: Commun Med (Lond). 2025 Nov 11;5:468. doi: 10.1038/s43856-025-01093-1 (PMC12618636; doi:10.1038/s43856-025-01093-1)
Supplement: Supplementary file 1 — Supplementary Information [file 43856_2025_1093_MOESM1_ESM.pdf]

SUPPLEMENTARY INFORMATION

A public health perspective of SARS-CoV-2 evolution and surveillance strategies in Germany from 2020 to 2023

Djin-Ye Oh[1,*,‡], Martin Hölzer[2,*], Daniela Börnigen[3], Sofia Paraskevopoulou[2], Susanne Duwe[1], Matthias Budt[1], Romy Kerber[4], Agata Mikolajewska[5], Sindy Böttcher[6], Janna Seifried[7], Walter Haas[4], Ralf Dürrwald[1], Stephan Fuchs[2], Stefan Kröger[4], Max von Kleist[3,8], Thorsten Wolff[1], and Martin Mielke[9] for the SARS-CoV-2 Diagnostics and Evolution Working Group at Robert Koch Institute[†]

[1]Influenza and Other Respiratory Viruses (FG17), Robert Koch Institute, Berlin, Germany

[2]Genome Competence Centre (MF1), Robert Koch Institute, Berlin, Germany

[3]Systems medicine of infectious diseases (P5), Robert Koch Institute, Berlin, Germany

[4]Respiratory Infections (FG36), Robert Koch Institute, Berlin, Germany

[5]Strategy and Incident Response (ZBS7), Robert Koch Institute, Berlin, Germany

[6]Gastroenteritis and Hepatitis Pathogens and Enteroviruses (FG15), Robert Koch Institute, Berlin, Germany

[7]Infectious Disease Epidemiology (Dept. 3), Robert Koch Institute, Berlin, Germany

[8]Department of Mathematics and Computer Science, Freie Universität Berlin, Germany

[9]Infectious Diseases (Dept. 1), Robert Koch Institute, Berlin, Germany

*These authors contributed equally to this work and share first authorship.

[†]A full list of members of the SARS-CoV-2 Diagnostics and Evolution Working Group at Robert Koch Institute is provided at the end of the Supplementary Information section.

[‡]Correspondence: OhD@rki.de

SUPPLEMENTARY TEXT T1

Genomic data collections and mutation frequencies

To illustrate the chronology of variant evolution between the start of the pandemic in 2020 and May 2023, two large sequence datasets were used: (i) all (as “randomly” selected) SARS-CoV-2 genomes from the German genomic surveillance data set obtained from the German Electronic Sequence Data Hub (DESH), which was operational for the nationwide collection of SARS-CoV-2 genomes from January, 2021 to May, 2023 (https://github.com/robert-koch-institut/SARS-CoV-2-Sequenzdaten_aus_Deutschland)[1,2]; and (ii) all international genomes published via GISAID[3-5]. Briefly, mutation profiles were generated on all high-quality genomes sampled through the end of May, 2023, namely 360,800 German sequences along with 8,598,137 GISAID sequences. Based on such retrieved mutation profiles (available online in the https://osf.io/9r4ws OSF repository at both nucleotide (nt) and amino acid (aa) level), the frequencies of all amino acid substitutions (aa mutations) were computed for each calendar week.

The analyses depicted in Fig. 2 were focused on mutations that appeared frequently across genomes, regardless of virus lineage. A mutation was defined as 'fixed' if it was nonsynonymous and appeared in at least 70 % of samples in any given calendar week. The GISAID sequence data dump[3] was obtained on 2023-06-19 (doi: 10.55876/gis8.240415ce) and the German (DESH) dataset was downloaded on 2023-06-17 (doi:10.5281/zenodo.8046538), while all genomes were selected until the end of May 2023 (sampling date). To minimize sampling bias in our study of the German genomic surveillance data set, we only included SARS-CoV-2 genome sequences that were explicitly labeled as "randomly sampled". This corresponds usually to a randomly selected sample that tested positive during PCR diagnostics. This approach ensures a more representative dataset and increases the reliability of results. Subsequently, high-quality genomes were extracted by counting the number of ambiguous "N" bases per genome and only those were kept with less than 100 "N", resulting in 360,800 German sequences and 8,598,137 international (GISAID) sequences. Next, covsonar (https://github.com/rki-mf1/covsonar) was used, a database-driven system for handling genomic sequences of SARS-CoV-2 and screening genomic profiles, to generate mutation profiles for all genomes, while the sampling calendar week (CW) for each sample was added to the resulting TSV with a custom script. Such retrieved mutation profiles were filtered using the "max 100 N" IDs and split into the respective calendar weeks, while mutation profiles were distinguished on nucleotide (nt) and amino acid (aa) levels. The resulting nt and aa mutation profiles per calendar week and the filtered genomes with less than 100 ambiguous N bases can be found in the OSF repository: https://osf.io/9r4ws.

Such filtered mutation profiles were then used to calculate the mutation frequencies per calendar week, independent of lineage assignments (script at https://github.com/hoelzer/sc2-consensus-mutation-profile (v0.0.1)). The aa changes and their frequencies above 70 % and per calendar week were summarized for plotting, while all aa changes with a frequency higher than 70 % in at least one CW were included. The lineage proportions were also summarized per calendar week from the same files for plotting (see https://osf.io/9r4ws).

To visualize the frequencies of aa changes for each calendar week between week 05/2020 and week 22/2023 in Germany as well as in an international context the R packages *ggplots* and *ComplexHeatmaps* were used.

SUPPLEMENTARY REFERENCES FOR TEXT T1

1. Oh, D. Y. *et al.* Advancing Precision Vaccinology by Molecular and Genomic Surveillance of Severe Acute Respiratory Syndrome Coronavirus 2 in Germany, 2021. *Clin Infect Dis* **75**, S110-S120 (2022). https://doi.org/10.1093/cid/ciac399
2. Robert Koch-Institut. SARS-CoV-2 Sequenzdaten aus Deutschland (2023-06-16). *Zenodo* (2023). https://doi.org/https://doi.org/10.5281/zenodo.8046538
3. Khare, S. *et al.* GISAID's Role in Pandemic Response. *China CDC Wkly* **3**, 1049-1051 (2021). https://doi.org/10.46234/ccdcw2021.255
4. Elbe, S. & Buckland-Merrett, G. Data, disease and diplomacy: GISAID's innovative contribution to global health. *Glob Chall* **1**, 33-46 (2017). https://doi.org/10.1002/gch2.1018
5. Shu, Y. & McCauley, J. GISAID: Global initiative on sharing all influenza data - from vision to reality. *Euro Surveill* **22** (2017). https://doi.org/10.2807/1560-7917.ES.2017.22.13.30494

SUPPLEMENTARY FIGURES

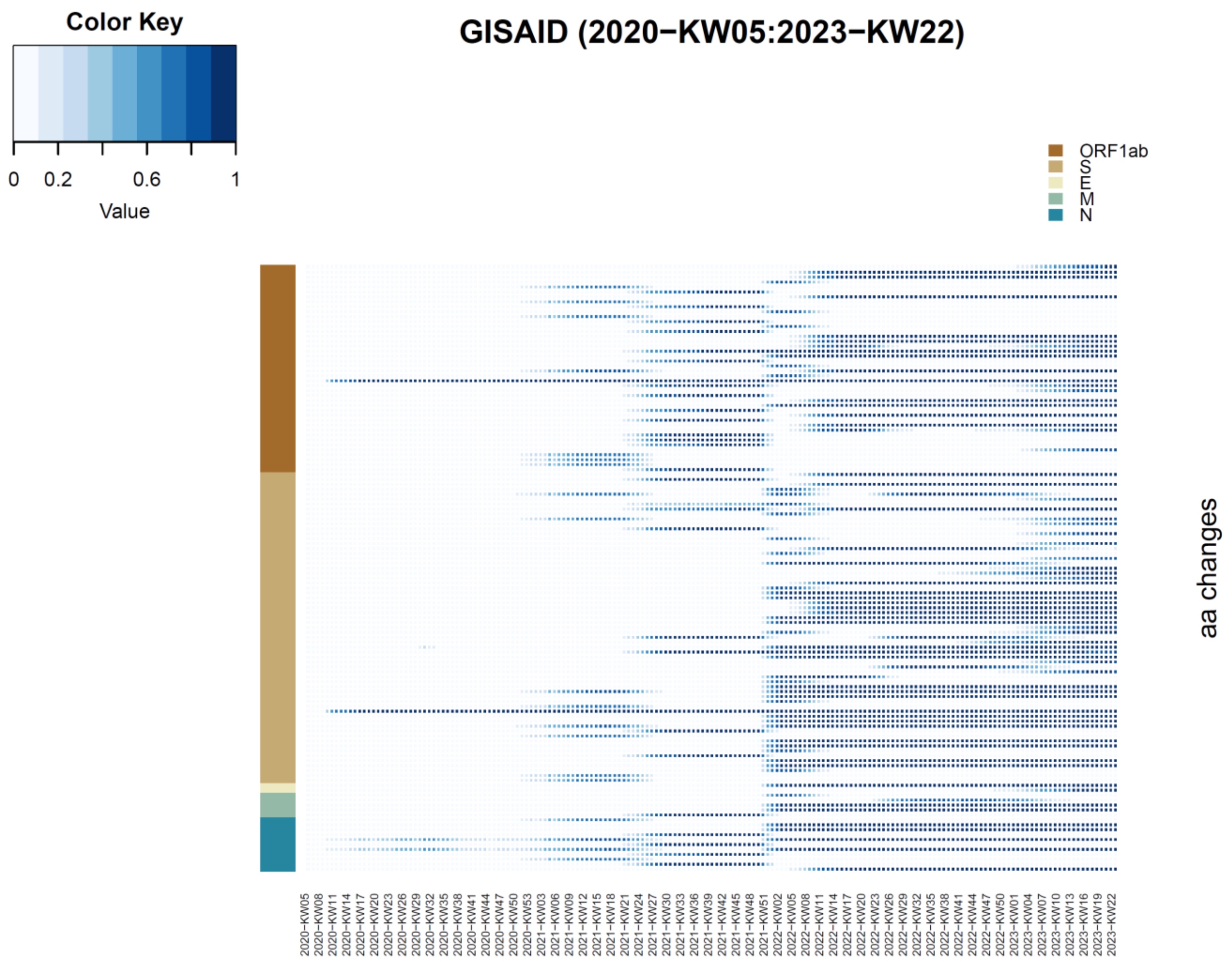

Supplementary Figure S1: Global view of mutation frequencies in the SARS-CoV-2 genome between beginning of 2020 and mid of 2023. Amino acid (aa) substitutions shown here have exceeded a frequency of 70 % in at least one calendar week (see **Supplementary Text** for further methodological details).

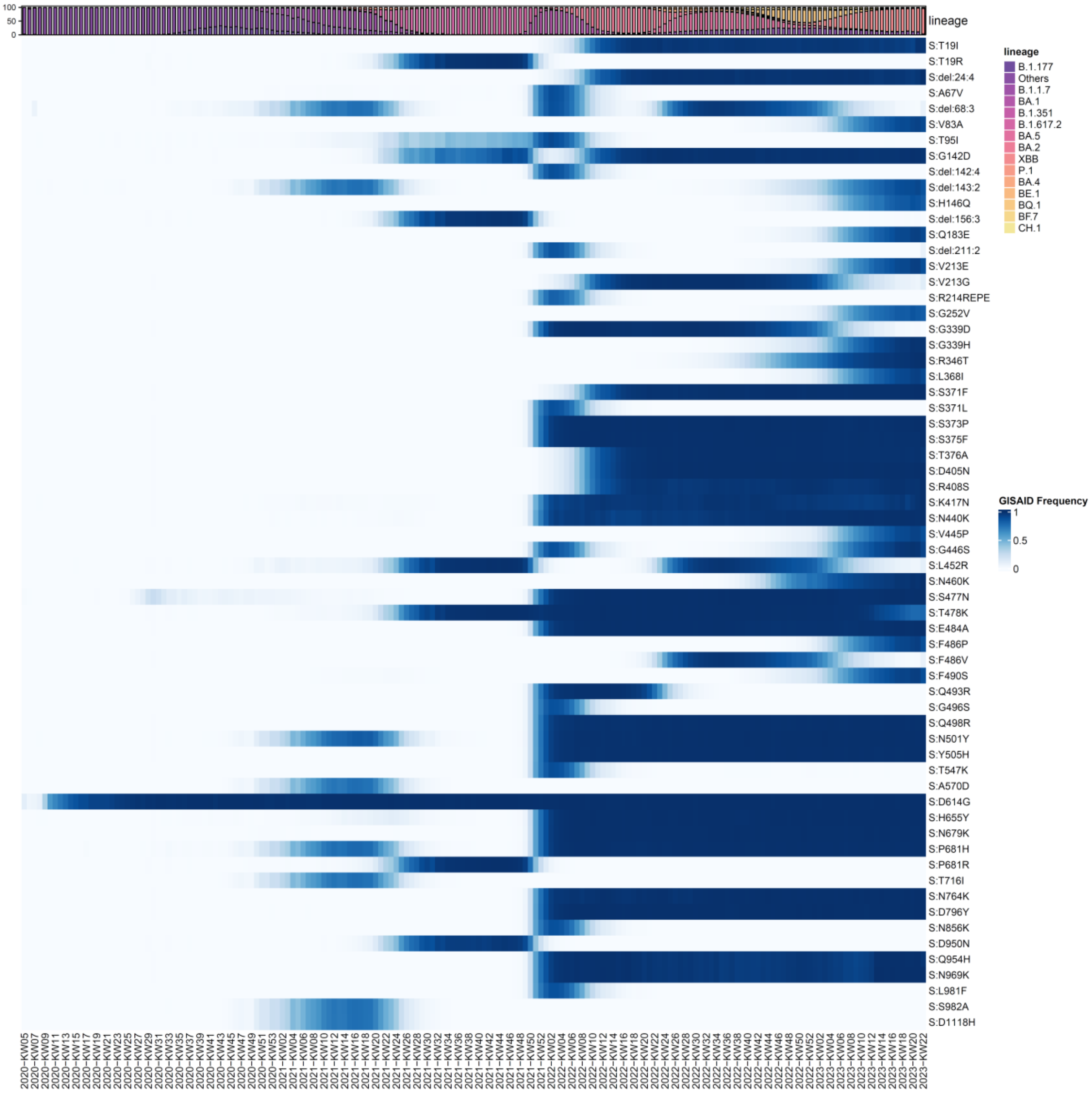

Supplementary Figure S2: Mutation frequencies in the S protein of the SARS-CoV-2 genome in GISAID (international) between beginning of 2020 and mid of 2023. Amino acid (aa) substitutions shown here have exceeded a frequency of 70 % in at least one calendar week (see **Supplementary Text** for further methodological details). The abundance of selected major lineages circulating on international level during that time is visualized at the top of the heatmap.

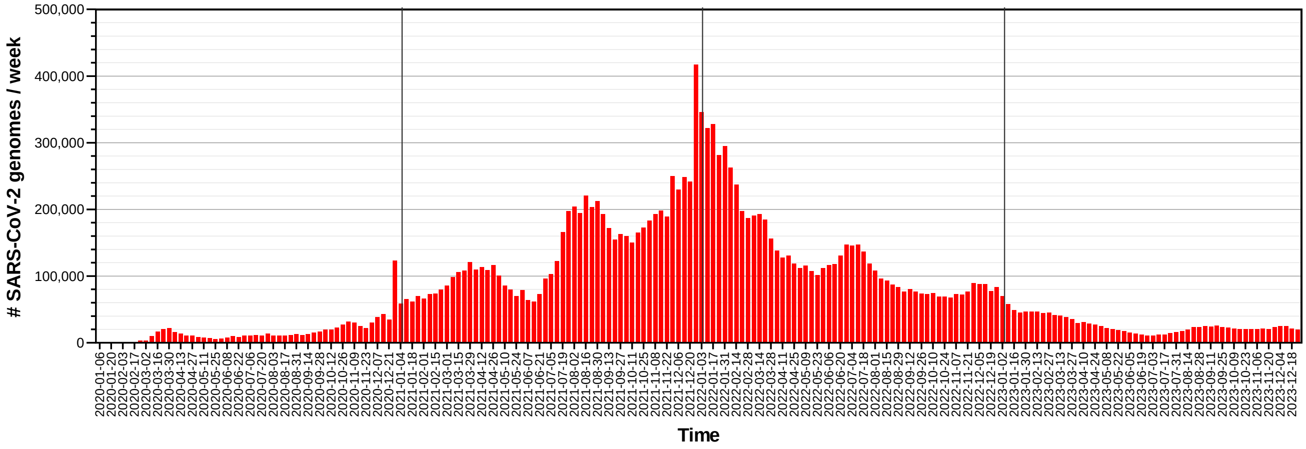

Supplementary Figure S3: Weekly number of SARS-CoV-2 genome sequences submitted to the GISAID Repository, mid-2020 to end-2023.

SUPPLEMENTARY TABLES

Supplementary Table S1: WHO-designated Omicron variants of interest and variants under monitoring (VOIs and VUMs), including their most relevant mutations and lineage properties, between May 2023 and December 2024.

Lineage	WHO designation	Time period	Relevant spike mutations	Lineage properties
XBB.1.5	Variant of Interest (VOI)	Jan 2023 – Jun 2024	XBB.1 + F486P	- Immune escape properties (similar to XBB.1) [1,2]
XBB.1.16		Apr 2023 – Jun 2024	XBB.1 + E180V, T478R, F486P	- Binding affinity of RBD to ACE2 is lower than that of XBB.1.5 RBD & infectivity similar to XBB.1.5 [3]<br>- Immune escape properties (similar to XBB.1 and XBB.1.5) [4]
EG.5		Aug 2023 – Jun 2024 (VUM since Jul 2023)	XBB.1.9.2 + F456L	- Immune escape properties [5]<br>- EG.5.1 showed high epidemic spread compared to outcompeted XBB subvariants (including XBB.1.5), owing to increased capacity to evade the humoral immunity induced by XBB breakthrough infections [6]
BA.2.86		Nov 2023 – Nov 2024 (VUM since Aug 2023)	BA.2 + R21T, S50L, 69_70del, V127F, F157S, R158G, 211del, L212I, L216F, H245N, A264D, I332V, K356T, V445H, N450D, N481K, A484K, 483del	- Limited immune escape relative to concurrently circulating variants at that time (compared to XBB.1.5 which was globally dominant before) [7-9]<br>- Antigenically distinct from XBB.1.5 and previous Omicron variants, and can evade XBB-induced and XBB-effective NAbs targeting various epitopes [10]<br>- Compared to EG.5.1, BA.2.86 i) is more transmissible, ii) has comparable drug sensitivity, and iii) has lower replication efficiency [6]<br>- Highly immunoevasive, evading BA.1- and BA.5-vaccine-induced humoral immunity [8]
JN.1		Dec 2023 – today	BA.2.86 + L455S	- Increased transmissibilty [11]<br>- Increased immune evasion relative to cocirculating variants [12-14]<br>- Higher R_e than BA.2.86.1 and HK.3, leading JN.1 to become dominant worldwide[11]<br>- Showed robust resistance to monovalent XBB.1.5 vaccine sera compared with BA.2.86[14]
BA.2.75		Jul 2022 – Oct 2023	BA.2 + K147E, W152R, F157L, I210V, G257S,	- Higher ACE-binding affinity than BA.4/BA.5<br>- Strong humoral immune evasion and both RBD- and

			D339H, G446S, N460K, Q493R reversion (or R493Q)	NTD-targeting antibody escape [15]
KP.2		Apr 2024 – today	JN.1 + R346T, F456L, V1104L	- Evades neutralization with greater efficiency than JN.1[16]<br>- Declared as VUM owing to concerning Spike mutations additional to the parental ones from JN.1, and due to its widespread detection; however, clear evidence of a transmission / growth advantage is not available.
KP.3	Variant under Monitoring (VUM)	Apr 2024 – today	JN.1 + F456L, Q493E, V1104L	- Significant increase in ACE2-Spike binding affinity compared with JN.1 [17]<br>- Evades neutralization with greater efficiency than JN.1[16]<br>- Declared as VUM owing to concerning Spike mutations additional to the parental ones from JN.1, and due to its widespread detection; however, clear evidence of a transmission / growth advantage is not available.
KP.3.1.1		Jul 2024 – today	KP.3 + S31del	- Significant increase in ACE2-Spike binding affinity compared with JN.1 [16,17]<br>- Higher R_e than JN.1, KP.2, and KP.3 [18]<br>- Higher pseudovirus infectivity and neutralization activity than KP.3 [18]<br>- Declared as VUM owing to concerning Spike mutations additional to the parental ones from JN.1, and due to its widespread detection; however, clear evidence of a transmission / growth advantage is not available.
XEC		Sep 2024 – today	JN.1 + T22N, F59S, F456L, Q493E, V1104L	- Growth advantage compared to currently circulating SARS-CoV-2 variants, e.g. KP.3.1.1 [12]<br>- Limited immune evasion, relative to co-circulating variants and from JN.1 or KP.2 mRNA booster vaccines [12]<br>- Enhanced humoral immune evasion and RBD-targeting antibody escape capabilities [12]<br>- Significant increase in ACE2-Spike binding affinity compared with JN.1 [17]<br>- Enhanced humoral immune evasion and RBD-targeting Ab escape [17]

Supplementary Table S2: All used GISAID data is available through the GISAID identifier "EPI_SET_240415ce" and the corresponding doi: 10.55876/gis8.240415ce

SUPPLEMENTARY REFERENCES FOR TABLE S1

1 World Health Organization. XBB.1.5 Updated Risk Assessment, 20 June 2023. *https://www.who.int/docs/default-source/coronaviruse/20230620xbb.1.5.pdf* (2023).

2 Yajima, H. *et al.* Molecular and structural insights into SARS-CoV-2 evolution: from BA.2 to XBB subvariants. *mBio* **15**, e0322023 (2024). https://doi.org/10.1128/mbio.03220-23

3 Yamasoba, D. *et al.* Virological characteristics of the SARS-CoV-2 omicron XBB.1.16 variant. *The Lancet. Infectious diseases* **23**, 655-656 (2023). https://doi.org/10.1016/S1473-3099(23)00278-5

4 World Health Organization. XBB.1.16 Updated Risk Assessment, 05 June 2023. *https://www.who.int/docs/default-source/coronaviruse/05062023xbb.1.16.pdf* (2023).

5 World Health Organization. Updated Risk Evaluation for EG.5 and its sublineages, 21 November 2023. *https://www.who.int/docs/default-source/coronaviruse/21112023_eg.5_ure.pdf* (2023).

6 Tamura, T. *et al.* Virological characteristics of the SARS-CoV-2 BA.2.86 variant. *Cell Host Microbe* **32**, 170-180 e112 (2024). https://doi.org/10.1016/j.chom.2024.01.001

7 CDC. Update on SARS-CoV-2 Variant BA.2.86 *https://www.cdc.gov/ncird/whats-new/covid-19-variant-update-2023-11-27.html* (2023).

8 Uriu, K. *et al.* Transmissibility, infectivity, and immune evasion of the SARS-CoV-2 BA.2.86 variant. *The Lancet. Infectious diseases* **23**, e460-e461 (2023). https://doi.org/10.1016/S1473-3099(23)00575-3

9 World Health Organization. Initial Risk Evaluation of BA.2.86 and its sublineages, 21 November 2023. *https://www.who.int/docs/default-source/coronaviruse/21112023_ba.2.86_ire.pdf* (2023).

10 Yang, S. *et al.* Antigenicity and infectivity characterisation of SARS-CoV-2 BA.2.86. *The Lancet. Infectious diseases* **23**, e457-e459 (2023). https://doi.org/10.1016/S1473-3099(23)00573-X

11 World Health Organization. Updated Risk Evaluation of JN.1, 15 April 2024. *https://www.who.int/docs/default-source/coronaviruse/15042024_jn1_ure.pdf* (2024).

12 World Health Organization. Initial Risk Evaluation of XEC, 09 December 2024. *https://www.who.int/docs/default-source/coronaviruse/09122024_xec_ire.pdf* (2024).

13 Wang, Q. *et al.* Recurrent SARS-CoV-2 spike mutations confer growth advantages to select JN.1 sublineages. *Emerg Microbes Infect* **13**, 2402880 (2024). https://doi.org/10.1080/22221751.2024.2402880

14 Kaku, Y. *et al.* Virological characteristics of the SARS-CoV-2 JN.1 variant. *The Lancet. Infectious diseases* **24**, e82 (2024). https://doi.org/10.1016/S1473-3099(23)00813-7

15 Cao, Y. *et al.* Characterization of the enhanced infectivity and antibody evasion of Omicron BA.2.75. *Cell Host Microbe* **30**, 1527-1539 e1525 (2022). https://doi.org/10.1016/j.chom.2022.09.018

16 Chen, N. *et al.* Comparative Analysis of Host Cell Entry Efficiency and Neutralization Sensitivity of Emerging SARS-CoV-2 Lineages KP.2, KP.2.3, KP.3, and LB.1. *Vaccines (Basel)* **12** (2024). https://doi.org/10.3390/vaccines12111236

17 Liu, J. *et al.* Enhanced immune evasion of SARS-CoV-2 variants KP.3.1.1 and XEC through N-terminal domain mutations. *The Lancet. Infectious diseases* **25**, e6-e7 (2025). https://doi.org/10.1016/S1473-3099(24)00738-2

18 Kaku, Y. *et al.* Virological characteristics of the SARS-CoV-2 KP.3.1.1 variant. *The Lancet. Infectious diseases* **24**, e609 (2024). https://doi.org/10.1016/S1473-3099(24)00505-X

MEMBERS OF THE SARS-COV-2 DIAGNOSTICS AND EVOLUTION WORKING GROUP AT ROBERT KOCH INSTITUTE (RKI)

Daniela Börnigen	Systems medicine of infectious diseases (P5), RKI
Sindy Böttcher	Gastroenteritis and Hepatitis Pathogens and Enteroviruses (FG15), RKI
Matthias Budt	Influenza and Other Respiratory Viruses (FG17), RKI
Sébastien Calvignac-Spencer	Helmholtz Institute for One Health, Helmholtz-Centre for Infection Research, Greifswald, Germany
Ralf Dürrwald	Influenza and Other Respiratory Viruses (FG17), RKI
Susanne Duwe	Influenza and Other Respiratory Viruses (FG17), RKI
Stephan Fuchs	Genome Competence Center (MF1), RKI
Walter Haas	Respiratory Infections (FG36), RKI
Martin Hölzer	Genome Competence Center (MF1), RKI
Matthew Huska	Genome Competence Center (MF1), RKI
Romy Kerber	Respiratory Infections (FG36), RKI
Stefan Kröger	Respiratory Infections (FG36), RKI
Marie Lataretu	Genome Competence Center (MF1), RKI
Vanda Marujo	Hospital Hygiene, Infection Prevention and Control (FG14), RKI
Janine Michel	Highly Pathogenic Viruses (ZBS1), RKI
Martin Mielke	Infectious Diseases (Dept. 1), RKI
Agata Mikolajewska	Strategy and Incident Response (ZBS7), RKI
Michaela Niebank	Strategy and Incident Response (ZBS7), RKI
Andreas Nitsche	Highly Pathogenic Viruses (ZBS1), RKI
Djin-Ye Oh	Influenza and Other Respiratory Viruses (FG17), RKI
Sofia Paraskevopoulou	Genome Competence Center (MF1), RKI
Aleksandar Radonic	Genome Competence Center (MF1), RKI
Janna Seifried	Infectious Disease Epidemiology (Dept. 3), RKI
Torsten Semmler	Genome Competence Center (MF1), RKI
Maureen Rebecca Smith	Systems medicine of infectious diseases (P5), RKI
Andrea Thürmer	Methods Development and Research Infrastructure (Dept. MFI), RKI
Wiep van der Toorn	Systems medicine of infectious diseases (P5), RKI; Dept. of Mathematics & Computer Science, Freie Universität Berlin, Germany
Max von Kleist	Systems medicine of infectious diseases (P5), RKI; Dept. of Mathematics & Computer Science, Freie Universität Berlin, Germany
Thorsten Wolff	Influenza and Other Respiratory Viruses (FG17), RKI
